# Use of the Neuman Systems Model in women's health: a scoping review

**DOI:** 10.1590/1980-220X-REEUSP-2025-0460en

**Published:** 2026-09-28

**Authors:** Nathaly Bianka Moraes Fróes, Dayane dos Reis Araújo Rocha Holanda, Samila Gomes Ribeiro, Paula Renata Amorim Lessa Soares, Francisca Elisângela Teixeira Lima, Viviane Martins da Silva, Marcos Venícios de Oliveira Lopes

**Affiliations:** 1 Universidade Federal do Ceará, Fortaleza, CE, Brazil.

**Keywords:** Models, Theoretical, Women’s Health, Nursing Theory, Scoping Review

## Abstract

**Objective:**

To map the scientific evidence on the use of the Neuman Systems Model (NSM) in the field of women’s health.

**Method:**

A scoping review. The search was conducted from April to June 2024 in the Scopus, PubMed, Embase, Ovid, Web of Science, CINAHL, *Biblioteca Virtual em Saúde, Biblioteca Digital Brasileira de Teses e Dissertações*, ProQuest, and Google Scholar databases. Primary studies, reviews, editorials, opinion articles, letters, comments, and grey literature that used the model in women’s health were included.

**Results:**

The model was used to assess stress related to procedures and motherhood, understand the experiences of patients with breast cancer, analyze violence, provide contraceptive counseling, and deliver health education regarding the female athlete triad. The model elements were identified in each setting studied; however, few studies used the model comprehensively.

**Conclusion:**

NSM has provided a framework for practice in women’s health, using the five variables to guide interventions directed toward health problems, strengthening lines of defense and resistance. It demonstrates potential as a theoretical framework in this field.

## INTRODUCTION

The Neuman Systems Model (NSM) is based on the philosophy that every human being is an open system affected by internal and external environments in ways that promote balance and adaptation^(1)^. It focuses on the response of the normal line of defense and lines of resistance to environmental stressors, as well as on the use of preventive nursing interventions to promote, attain, and maintain optimal wellness of the client system^(2)^.

The client system core is the central focus, whose priority is to maintain this basic structure free from stress. The lines of resistance and defense protect the system against stressors through the resources of physiological, psychological, sociological, developmental, and spiritual variables, which are activated when a stressor penetrates one of these lines, compromising system stability^(2)^.

The stressors against which the lines of defense and resistance protect the client system may be intrapersonal (within the client system), interpersonal (close to the boundaries of the client system), or extrapersonal (outside the client system). These stressors may be multiple, and their impact is influenced by the type, timing, and response of the client system, encompassing physiological, psychological, sociological, developmental, and spiritual variables^(3)^. Preventive actions, including primary, secondary, and tertiary interventions, are developed according to the timing and severity of the damage caused by these stressors to the lines of defense and resistance^(3)^. Figure 1 presents a graphical representation of NSM.

**Figure 1:**
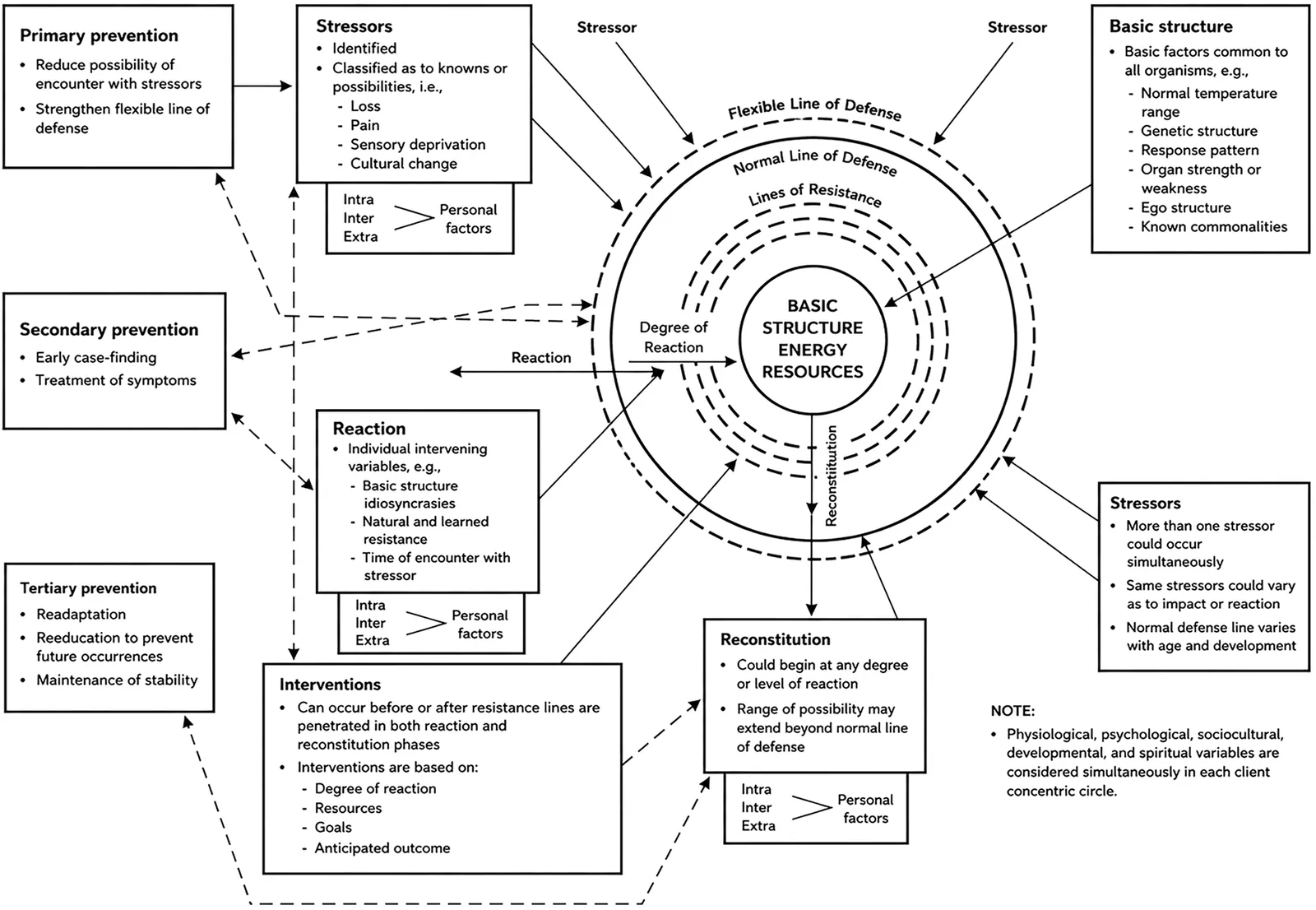
Neuman systems model diagram.

The use of NSM is necessary for testing various phenomena in the field of nursing within cultural and social environments^(4)^. In this regard, linking NSM to women’s health is promising because this field has expanded beyond a focus on maternal care to include sexual health, contraception, chronic conditions specific to women, interpersonal violence, and other phenomena^(5)^.

Women are the primary users of healthcare services and are the most vulnerable to imposed social stigmas, violence, rigid social norms, and social and economic disadvantages. Therefore, they are more susceptible to risks that may adversely affect their health^(6)^. At different stages of life, women are exposed to stressors that include psychological, social, and economic factors, which significantly influence health outcomes.

In this context, the complexity of women’s healthcare becomes evident. Therefore, nursing professionals play a crucial role in providing services aimed at disease prevention, health protection, and health promotion during prenatal consultations, gynecological preventive care visits, postpartum care, family planning services, and other spontaneous healthcare demands^(7)^.

Thus, for structured and effective nursing care focused on women’s health, the use of theories and models is essential for translating nursing knowledge into practice and research, as they provide a solid foundation for implementing and assessing interventions, promoting integration between theoretical knowledge and practical needs^(8)^.

Considering this information, NSM is appropriate for comprehensive care of women because it proposes an open-systems approach involving five major variables. It can be applied to a variety of populations and conditions and is uniquely adaptable to a wide range of nursing issues. Furthermore, it can be used to define the nurse’s primary, secondary, and tertiary roles within the client system^(9,10)^.

However, despite its explanatory potential and recognized applicability, the use of the model in women’s health still lacks systematization and critical depth. In many studies, the theoretical framework is used only partially, in a merely descriptive or illustrative manner, without full incorporation of its elements or explicit clarification of how these elements guide data analysis, interpretation of findings, or intervention planning. Such fragmented use may weaken the theoretical consistency of investigations and limit the cumulative advancement of knowledge in the field.

A search was conducted in the Cumulative Index to Nursing and Allied Health Literature (CINAHL Plus), PubMed/MEDLINE, and Scopus databases, as well as in the *Biblioteca Virtual em Saúde* (BVS) portal and the JBI Evidence Synthesis, Cochrane Database of Systematic Reviews, and Open Science Framework (OSF) Registry sources on April 28, 2024. However, no scoping or systematic reviews on the use of NSM in women’s health were identified.

Given this scenario, it becomes essential to critically map the scientific evidence on the use of NSM in women’s health, analyzing not only where it has been applied, but also how its elements have been incorporated. This initiative contributes to the epistemological strengthening of nursing, to the qualification of scientific production, and to the promotion of more coherent, grounded, and theoretically consistent care. Due to the holistic approach of NSM and its potential to address specific women’s health problems, and the scarcity of studies on its application in this area, the following question arose: how has NSM been used in the field of women’s health?

In this context, the objective is to map the scientific evidence on the use of NSM in women’s health. It is expected that the results of this review will contribute to a better understanding of NSM use in this setting.

## METHOD

### Study Design

This is a scoping review conducted in accordance with the JBI Manual for Evidence Synthesis and reported following the recommendations of the Preferred Reporting Items for Systematic Reviews and Meta-Analyses extension for Scoping Reviews (PRISMA-ScR)^(11,12)^. The review protocol is available on the OSF at: https://osf.io/zw52h/.

### Review Question

To guide the development of the review, the research question was structured using the PCC acronym, which stands for Population, Concept, and Context. Accordingly, the Population was considered open; the Concept included NSM; and the Context included women’s health. Thus, the following review question was established: how has NSM been used in the field of women’s health?

### Inclusion Criteria

Studies that used NSM, addressed the context of women’s health, and were published as primary studies, literature reviews, editorials, opinion articles, letters, comments, books, theses, or dissertations were included.

### Exclusion Criteria

Studies in which data regarding women’s health were not analyzed separately from those of other populations were excluded.

### Information Sources

The following databases were searched: Scopus (Elsevier), PubMed/MEDLINE, Embase, Ovid, Web of Science, CINAHL with Full Text, and the BVS portal. Moreover, grey literature was searched through the *Biblioteca Digital Brasileira de Teses e Dissertações*, ProQuest for international theses and dissertations, and Google Scholar (first ten pages).

A search of the reference lists of articles included in the final sample was also performed using the online tool ResearchRabbit.

### Search Strategy

Before conducting the database searches, a search was carried out in PubMed/MEDLINE to define the descriptors used in the strategy with the assistance of a librarian. Because NSM is not included in the Medical Subject Headings (MeSH), uncontrolled descriptors were used for databases adopting this vocabulary, based on terms identified in the titles, abstracts, and keywords of articles found during the preliminary search. Descriptors related to the concept of “women’s health” were identified in MeSH together with their synonyms. For the remaining databases, a search strategy adapted to the specific vocabularies was developed.

Similar descriptors were used for the grey literature. Searches were conducted without restrictions regarding language or year of publication. The descriptors used in each database and alternative source, as well as the search strategies, are available in the repository SciELO Data, the access link is in the Data availability seccion of this paper. Chart 1 presents one of the strategies used in the Web of Science database.

**Chart 1:** Search strategy used in the Web of Science database for the review – Fortaleza, CE, 2024.

Databases	Search strategy
Web of Science	“Neuman Systems Model” OR “Betty Neuman Systems Model” OR “Neuman’s System Theory” OR “Neuman’s System” OR “Neuman’s Model” OR “Neuman’s framework” OR “Betty Neuman’s System” OR “Betty Neuman’s Systems Model” OR “Neuman systems theory” OR “Neumans System” OR “Neuman System’s” OR “Betty Neuman’s assumptions” OR “Betty Neuman Health Care Systems Model” OR “Betty Neuman” (All Fields) and “Women” OR “Women’s Health” OR “Reproductive Health” OR Contraception OR “Breast Neoplasms” OR “Health, Reproductive” OR “Inhibition of Fertilization” OR “Fertilization Inhibition” OR “Fertility Control” OR “Birth Control” OR “Contraceptive Methods” OR “Female Contraception” OR “Neoplasm, Breast” OR “Breast Tumors” OR “Mammary Cancer” OR “Malignant Neoplasm of Breast” OR “Cancer of Breast” OR “Human Mammary Carcinomas” OR “Human Mammary Neoplasm” OR “Human Mammary Neoplasms” OR “Breast Carcinoma” OR “Change of Life, Female” OR “Pregnancies” OR “Gestation” OR “Health, Sexual” (All Fields)

### Study Selection

For the management of references retrieved from the databases, the online tool Rayyan was used during the stages of duplicate removal, title and abstract screening, and full-text review^(13)^.

The search of studies in the databases and grey literature was conducted on April 29, 2024. The study selection process took place between April 30 and June 10, 2024. First, duplicate articles were removed. Next, titles and abstracts were screened. The remaining studies were read in full and carefully assessed to justify their inclusion in or exclusion from the final sample. The title and abstract screening and full-text review stages were conducted by two reviewers. During the title and abstract screening stage, no disagreements occurred; however, during the full-text review stage, five disagreements arose, for which a third reviewer made the final decision regarding article inclusion. An electronic checklist was used to guide these selection stages (data repository link at the data availability section).

After the inclusion of studies in the final sample, the online tool ResearchRabbit was used to search the reference lists in order to identify additional studies that could answer the review question.

### Data Extraction

Data were extracted using an instrument developed by the authors, containing the following information: author(s); year of publication; country of origin (location where the source was conducted); objectives/purpose; population and sample size within the source of evidence (if applicable); population characteristics and inclusion and exclusion criteria; methods (study design, setting, duration, recruitment/selection, and data analysis); details of the intervention and comparator (if applicable); duration of the intervention (if applicable); details of the instrument used (if applicable); and results (if applicable).

In addition to the general characteristics, information relevant to the study objective was collected, including: women’s health area addressed; variables examined (physiological, psychological, sociocultural, developmental, and spiritual); environment (internal, external, and created); stressors (intrapersonal, interpersonal, and extrapersonal); affected lines of defense (flexible, normal, and resistance); prevention (primary, secondary, and tertiary); study limitations; conclusions; and reviewers’ comments.

### Evidence Analysis

Data regarding the general characteristics of the studies were analyzed using descriptive statistics (absolute and relative frequencies). Data related to the study objective were analyzed based on the structure of NSM and coded according to its elements, such as identified variables, stressors, lines of defense, and types of prevention applied as interventions, as well as the manner in which the model was used in the study (assessment, theoretical framework, intervention, etc.).

## RESULTS

As shown in Figure 2, the database search yielded 343 studies, of which 120 were duplicates and were removed. The remaining 223 studies were screened through title and abstract review. Of these, 183 were excluded according to the predefined criteria. Moreover, 17 studies did not have the full text available, either because access or purchase links were unavailable or because the journal website was inactive. When data were unavailable, attempts were made to contact the journals and article authors via email; however, no responses were received. Thus, 23 studies were included for full review, of which one was not related to women’s health and five did not actually use NSM. Therefore, 17 studies were included in the review. The search of grey literature, Google Scholar, and the reference lists of articles yielded 557 studies. Of these, 129 were not related to women’s health and 426 did not use NSM. Therefore, two additional studies were included in the review, resulting in a final sample of 19 studies.

**Figure 2:**
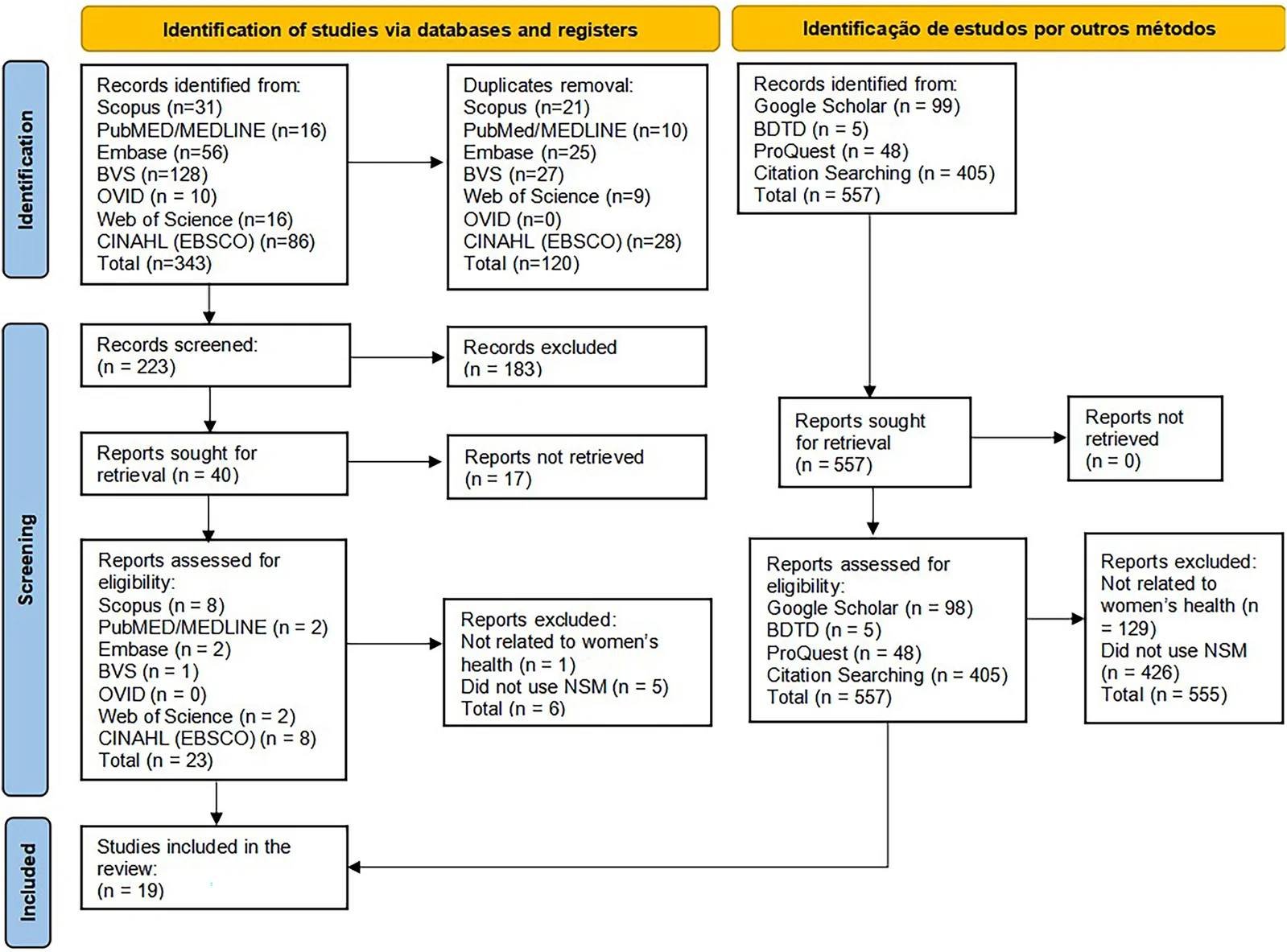
Preferred Reporting Items for Systematic Reviews and Meta-Analyses extension for Scoping Reviews flow diagram of study search and selection for the review^(12)^.

Chart 2 presents the characteristics of the studies included in the review according to study type, women’s health area, and objectives. Twelve studies were conducted in the United States, four in Türkiye, one in Sweden, one in South Korea, and one in Portugal. Methodological designs varied, with qualitative studies (n = 5), intervention studies (n = 3), and cross-sectional studies (n = 3) predominating. An expanded version of this table is available in the repository SciELO Data, the access link is in the Data availability seccion of this paper, the material details the relationship between the aspects examined in each article and the elements of NSM.

**Chart 2:** Characterization of the studies included in the review – Fortaleza, CE, 2024.

Author, year/country	Sample	Study design	Women’s health field	Objective
Aker and Özdemir, 2023/Turkey^(15)^	65 infertile women over 18 years old	Randomized clinical trial (pre and post-test)	Artificial insemination	Determine the effect of the Neuman Systems Model-based approach on stress and coping in women undergoing intrauterine insemination treatment.
Bigbee, 1984/United States^(16)^	Women	Narrative review	Maternal role	Review the relevant literature on the changing roles of women living in rural areas of the United States.
Bourdeanu and Dee, 2013/United States^(17)^	Women	Fictional case study	Breast cancer	Demonstrate the use of the Neuman Systems Model to assess and determine predictors of chemotherapy-induced nausea and vomiting in patients diagnosed with breast cancer.
Chaponniere, 2016/United States^(18)^	84 pregnant women aged 16 to 41 years old	Case series	Pregnancy and childbirth	Investigate the relationship between stress, stress buffers, acculturation, and birth outcomes of infants born to Mexican and Mexican-American mothers.
Doherty, 1997/United States^(19)^	Six women	Ethnographic study and focus groups	Domestic violence/spousal abuse	Explore spousal abuse in African American families living in Tucson, focusing on the experiences of abused women, in order to inductively form a cultural perspective.
Ïnan and Üstün, 2018/Turkey^(20)^	32 women aged 32 to 70 years old	Quasi-experimental pre and post-test	Breast cancer	Assess the effects of a home-based psychoeducational program on suffering, anxiety, depression, and quality of life in breast cancer survivors.
Ïnan and Üstün, 2016/Turkey^(21)^	14 women aged 33 to 68 years old	Descriptive qualitative	Breast cancer	Explain the experiences of Turkish breast cancer survivors in light of Neuman Systems Model.
Gigliotti, 1999/United States^(22)^	188 nursing students	Cross-sectional	Maternal role	Examine the conditions under which the stress of the student-mother role develops.
Gigliotti, 2007/United States^(23)^	69 university student mothers	Cross-sectional	Maternal role	Improve the external and internal validity of Gigliotti’s model of maternal role-student stress in middle-aged women.
Hemphill, 2005/United States^(24)^	17 homeless women aged 22 to 56	Descriptive qualitative	Violence	Engage women victims of violence experiencing homelessness in a dialogue that facilitates the discovery of their perception and personal strengths.
Kim et al., 2020/South Korea^(25)^	Single mothers	Integrative review	Maternal role	Comprehensively review domestic research on single mothers based on the main concepts that constitute Neuman Systems Model.
Lancaster, 2005/United States^(26)^	209 women	Correlational descriptive study	Breast cancer	Examine how women with a family history of breast cancer assess and cope with the risk of breast cancer.
Lindell and Olsson, 1991/Sweden^(27)^	Midwives	Theoretical study	Contraception	Explore how a Swedish midwife can practically use Neuman Systems Model in providing combined oral contraceptive counseling.
Mataoui, 2018/United States^(28)^	118 Arab women over 40 years of age	Cross-sectional	Breast cancer	Assess the well-being of the core population by identifying the screening rate for Arab Muslim women living in the United States aged 40 or older and 50 or older, and compare the results with data from the Centers for Disease Control and Prevention, which are 65.3% and 71.3%, respectively. Assess the interrelationships between acculturation, modesty, and knowledge about breast cancer screening. Assess the effects of stress on the core population.
Metin and Kulakaç, 2023/Turkey^(29)^	201 high-risk pregnant women	Methodological study	Pregnancy	Develop the Perceived Stress Scale in High-Risk Pregnancies based on Neuman Systems Model.
Reeves, 2004/United States^(30)^	Eight women victims of violence	Qualitative phenomenological	Intimate partner violence	Understand the childhood experiences of women who are victims of intimate partner violence.
Sabatini, 2003/United States^(31)^	11 women aged 16 to 27	Qualitative phenomenological	Pregnancy	Explore the meaning of the lived experience of teenage pregnancy for women who gave birth during adolescence.
Sousa et al., 2022/Portugal^(32)^	A 53-year-old woman	Qualitative case study	Breast cancer	Understand the experience of parental cancer of a mother in light of Neuman Systems Model, identify mothers’ needs, and prescribe nursing interventions using the International Classification for Nursing Practice taxonomy.
Tabone, 2013/United States^(14)^	45 female college athletes aged 18 to 22	Quasi-experimental pre and post-test	Female athlete triad	Identify knowledge gaps and highlight the importance of educating female university athletes about the female athlete triad. Provide education to female athletes about the prevalence of the female athlete triad.

Breast cancer and motherhood (pregnancy, childbirth, and the maternal role) were the most frequently addressed women’s health areas, with six studies each, followed by violence (domestic violence and intimate partner violence) (n = 3). Other topics, such as artificial insemination, contraception, and the female athlete triad, were also addressed.

NSM was used to improve or assess the effects of stress related to clinical procedures, pregnancy, and the maternal role. In the context of breast CA, patients’ experiences were analyzed, including aspects such as anxiety, depression, and quality of life. In addition, the physiological effects of chemotherapy treatment (nausea and vomiting) were also addressed.

Understanding the impact of violence on daily life and childhood experiences, as well as its cultural aspects, from the perspective of NSM, was also addressed in the studies included in this review. The model was also used in interventions involving contraceptive counseling provided by midwives and health education related to the female athlete triad. The female athlete triad is a syndrome composed of eating disorders, amenorrhea, and osteoporosis. The syndrome may occur in high-performance athletes with inadequate nutrition^(14)^.

Concerning the NSM elements used in the studies, it was observed that most studies (n = 14) used some classification of variables (physiological, psychological, sociocultural, developmental, and spiritual), whereas five (26.3%) did not (Chart 3). Among those that did, psychological, sociocultural, and developmental variables were the most prevalent (85.8%), followed by physiological (78.6%) a nd spiritual variables (71.4%).

**Chart 3:** Elements of the Neuman Systems Model used in the studies – Fortaleza, CE, 2024.

Author/year	Variables	Stressors	Lines of defense/resistance	Prevention
Aker and Özdemir, 2023^(15)^	Not classified	Intrapersonal Interpersonal Extrapersonal	Not addressed	Secondary
Bigbee, 1984^(16)^	Not classified	Intrapersonal Interpersonal Extrapersonal	FLD	Not addressed
Bourdeanu and Dee, 2013^(17)^	Physiological Psychological Sociocultural Developmental Spiritual	Intrapersonal Interpersonal Extrapersonal	Not addressed	Primary Secondary Tertiary
Chaponniere, 2016^(18)^	Physiological Psychological Sociocultural Developmental Spiritual	Not classified	FLD NLD LR	Not addressed
Doherty, 1997^(19)^	Not classified	Intrapersonal Interpersonal Extrapersonal	FLD LR	Primary Secondary
Ïnan and Üstün, 2018^(20)^	Not classified	Not classified	LR	Tertiary
Ïnan and Üstün, 2016^(21)^	Physiological Psychological Sociocultural Developmental Spiritual	Intrapersonal Interpersonal Extrapersonal	Not addressed	Tertiary
Gigliotti, 1999^(22)^	Physiological Psychological Sociocultural Developmental	Intrapersonal Interpersonal	FLD NLD	Not addressed
Gigliotti, 2007^(23)^	Psychological Sociocultural Developmental	Not classified	FLD LN	Not addressed
Hemphill, 2005^(24)^	Psychological Spiritual	Intrapersonal Interpersonal Extrapersonal	Not addressed	Not addressed
Kim et al., 2020^(25)^	Physiological Psychological Sociocultural Developmental Spiritual	Intrapersonal Interpersonal Extrapersonal	FLD NLD LR	Not addressed
Lancaster, 2005^(26)^	Not classified	Not classified	FLD	Primary
Lindell and Olsson, 1991^(27)^	Physiological Psychological Sociocultural Developmental Spiritual	Intrapersonal Interpersonal Extrapersonal	FLD	Primary
Mataoui, 2018^(28)^	Sociocultural Developmental	Not classified	NLD	Not applicable
Metin and Kulakaç, 2023^(29)^	Physiological Psychological Sociocultural Developmental Spiritual	Not classified	Not addressed	Not addressed
Reeves, 2004^(30)^	Physiological Psychological Sociocultural Developmental Spiritual	Intrapersonal Interpersonal Extrapersonal	FLD NLD LR	Primary Secondary Tertiary
Sabatini, 2003^(31)^	Physiological Psychological Sociocultural Developmental Spiritual	Not classified	Not addressed	Not addressed
Sousa et al., 2022^(32)^	Physiological Psychological Sociocultural Developmental Spiritual	Intrapersonal Interpersonal Extrapersonal	FLD LR	Primary Secondary Tertiary
Tabone, 2013^(14)^	Physiological	Not classified	Not addressed	Primary

Legend: FLD – Flexible Line of Defense; NLD – Normal Line of Defense; LR – Line of Resistance.

Stressors (intrapersonal, interpersonal, and extrapersonal) were classified in 11 studies (58%). The lines of defense (flexible and normal) and lines of resistance were addressed in 12 studies (63%); among these, three addressed all lines. The flexible line of defense, which is the first to come into contact with stressors, was the most frequently reported among studies that addressed this element (n = 7; 37%). Prevention as an intervention (primary, secondary, and tertiary) was implemented or suggested in ten articles. Three of these addressed all levels of prevention, with primary prevention being the most frequent (n = 7; 37%). However, it should be taken into consideration that each phenomenon has prevention levels that are more appropriate to the issue being addressed.

The environment, classified as internal, external, and created, was identified in two studies. In the first, a qualitative study involving homeless women who had experienced violence, the created environment consisted of the women’s perceptions of their personal responses to environmental stressors^(24)^. In the second study, an integrative review on the role of single mothers, the external environment was composed of parental support, support from the child’s father, adult attachment, and parental attachment^(25)^.

Overall, the use of NSM enabled the achievement of positive outcomes, such as reduced stress levels and improved coping mechanisms, enhanced organization and planning of nursing care through a holistic perspective, assessment of the relationships among the variables under study, and the possibility of being used in conjunction with other theories^(15,17,21,22,30,32)^.

## DISCUSSION

This review provided an overview of how NSM has been used in women’s health and its practical and theoretical applicability. Breast CA, the most frequently addressed topic, was examined from the woman’s perspective, considering the stress and anxiety associated with both the diagnosis and the risk of developing the disease. Identifying the variables involved in this issue is important for nursing care planning.

Not only psychological aspects but also physiological factors related to disease treatment were associated with stressors, including the type of treatment, other medical conditions, additional problems (e.g., fatigue), lifestyle habits, antidepressant use, and lack of preparation to cope with persistent symptoms^(21)^. This suggests that the model can be applied both to subjective situations, in qualitative studies, and to objective situations, in quantitative studies.

The process of becoming a mother, including pregnancy, childbirth, and the maternal role, was another aspect extensively explored using NSM, particularly with regard to the stress and anxiety inherent to this social role and life stage. The model appears to be appropriate for assessing complex situations involving multiple variables, such as the maternal role–student role, in which roles overlap and the effects of interpersonal stressors are of paramount importance^(22)^. A similar finding was reported in a study on the meaning of adolescent pregnancy, whose authors stated that NSM provides structure and organization to a topic that would otherwise be extremely complex^(31)^.

Violence, another complex topic, was explored from cultural and social-condition perspectives. Understanding, through NSM, how this phenomenon affected the lives of the women who participated in the studies highlighted the nurse’s role in planning interventions aimed at restoring the stability of these clients’ systems.

The elements of NSM were addressed in different ways. In a study that assessed the effects of a home-based psychoeducational program on distress, anxiety, depression, and quality of life among breast CA survivors, strengthening the lines of resistance through tertiary prevention was found to reduce psychological stress and improve the quality of life of these patients^(20)^.

A study that assessed violence against women found that tertiary prevention would be the most appropriate intervention, since previous stressors had already caused disturbances within the system. This can be achieved through social support and educational information addressing the long-term physical and psychological impact of intimate partner violence^(30)^.

Primary prevention, in turn, may be applied to topics related to health education, such as contraceptive counseling, whose objective is to prevent the risks of a stressor threatening the core structure, such as an unintended pregnancy, and to strengthen the flexible line of defense^(27)^. To achieve this, clients’ perceptions must be assessed before the development of a specific intervention. The intervention should reduce system stress and enhance individual and collective positive health factors, thereby strengthening the client system^(24)^.

The findings indicate that the elements of NSM are applicable to women’s health in a variety of situations. However, few studies explored the environment in their analyses. It is important that this element be taken into consideration, since the way a woman expresses the created environment depends on the perceived resources she brings to a particular experience, and the perception a woman creates about an experience may influence her health status^(24)^.

Considering the wide range of situations and methodological designs in which NSM has been used in the context of women’s health, implementation of the model by nurses may increase the effectiveness of care by identifying problems and promoting positive changes^(21)^. Furthermore, the model enables participants to become active members of the care process, in which self-perception and self-assessment are regarded as essential aspects for achieving and maintaining well-being^(31)^.

The studies also highlighted the benefits of the holistic view of women provided by the model. Neuman states that nursing practice is holistic and that including the client’s perception of health is an integral part of care delivery. Through this model, the entire health process is addressed, including health promotion, acute care, chronic care, and end-of-life care^(24,30)^. Determining the existing levels of positive energy resources available for defense against stressors helps women in their pursuit of well-being. Nurses are responsible for assessing these energy resources and using them to develop interventions that minimize the impact of stressors on the system, promoting the restoration of health at the highest possible level^(24)^.

The model has the potential to be used as a theoretical framework in the areas of women’s health addressed in this review and, possibly, in others that were not identified among the findings of this study. Women’s health is multidimensional, and a model that considers not only the client’s individual aspects but also her interpersonal relationships and extrapersonal factors provides a holistic perspective. This approach broadens the range of interventions available to nurses working with this population, enabling more comprehensive and effective care.

### Study Limitations

A considerable number of studies could not be retrieved because they consisted of theses or dissertations that were unavailable due to the policies of the institutions responsible for their repositories.

## CONCLUSION

NSM has been used in Women’s Health as a framework to assess and intervene in the health of women with breast CA; assess stress related to the maternal role; reduce stress and anxiety associated with procedures; understand the impact of different forms of violence; develop primary prevention strategies, such as health education for contraceptive counseling; and provide clarification regarding conditions inherent to women’s health. The approach adopted using NSM in the studies was predominantly qualitative, addressing complex and subjective topics involving multiple elements that require in-depth assessment by nurses.

Although it is a holistic model, few studies used all of its elements within their framework. Among the variables, the most frequently used were psychological, sociocultural, and developmental variables. Stressors, lines of defense, and prevention as intervention were addressed in similar proportions in slightly more than half of the studies. The environment, on the other hand, was the least explored element.

The decision not to use all elements of the model may be related to the specific situations addressed in each study, as authors may not have considered their complete application necessary. The limited focus on the environment may be associated with a perspective more centered on the problem than on the context, potentially underestimating the scope of the aspects analyzed in studies that did not incorporate this element. This highlights the need for additional studies in the field of women’s health that make use of the model’s potential to improve women’s health by including both the external and internal environments and ensuring that the client system core remains free from, or less exposed to, stress.

As demonstrated in this study, NSM offers a holistic and comprehensive perspective that addresses not only individual needs but also the entire context in which women are situated. Through its elements, the model may be applied comprehensively or in specific situations, providing a broad perspective and facilitating the identification of intervention points aimed at achieving client system stability. This application may occur through analysis based on the model itself or through middle-range theories and situation-specific theories derived from NSM, since it is a theory with a higher level of abstraction.

This study reinforces the potential of NSM as an organizing framework for care, supporting interventions aimed at reducing stressors and strengthening women’s protective mechanisms, in alignment with a systemic and comprehensive perspective of women’s health. Furthermore, by identifying areas that have already been explored and contexts that remain underinvestigated, this study contributes to the establishment of future research agendas, encouraging investigations that apply the model more robustly and expand its use in public policies, Primary Health Care, and health promotion strategies.

## Data Availability

The entire dataset supporting the findings of this study is in the repository SciELO Data under the link https://doi.org/10.48331/SCIELODATA.DP0S60.
