# Supplementary material for: Use of the Neuman Systems Model in women's health: a scoping review
Source: Rev Esc Enferm USP. 2026 Sep 28;60:e20250460. doi: 10.1590/1980-220X-REEUSP-2025-0460en (PMC13619120; doi:10.1590/1980-220X-REEUSP-2025-0460en)
Supplement: Translation

# Translation: Uso do Modelo de Sistemas de Neuman na saúde da mulher: revisão de escopo

**DOI:** 10.1590/1980-220X-REEUSP-2025-0460pt

**Authors:** Nathaly Bianka Moraes Fróes, Dayane dos Reis Araújo Rocha Holanda, Samila Gomes Ribeiro, Paula Renata Amorim Lessa Soares, Francisca Elisângela Teixeira Lima, Viviane Martins da Silva, Marcos Venícios de Oliveira Lopes

**Affiliations:** 1 Universidade Federal do Ceará, Fortaleza, CE, Brasil.

**Keywords:** Modelos Teóricos, Saúde da Mulher, Teoria de Enfermagem, Revisão de Escopo

## Abstract

**Objetivo:**

Mapear as evidências científicas sobre o uso do Modelo de Sistemas de Neuman (MSN) na área da saúde da mulher.

**Método:**

Revisão de escopo. A busca foi realizada de abril a junho de 2024 nas bases de dados Scopus, PubMed, Embase, Ovid, *Web of Science*, CINAHL, Biblioteca Virtual em Saúde, Biblioteca Digital Brasileira de Teses e Dissertações, ProQuest e *Google Scholar*. Incluíram-se pesquisas primárias, revisões, editoriais, artigos de opinião, cartas, comentários e literatura cinzenta, que utilizaram o modelo na saúde da mulher.

**Resultados:**

O modelo foi utilizado para avaliar o estresse relacionado a procedimentos e à maternidade, compreender as experiências de pacientes com câncer de mama, analisar a violência, oferecer aconselhamento contraceptivo e educar sobre saúde na tríade da atleta feminina. Os elementos do modelo foram identificados em cada cenário estudado, porém poucos utilizaram de forma completa.

**Conclusão:**

O MSN tem fornecido estrutura para atuação na saúde feminina, utilizando-se as cinco variáveis para traçar as intervenções direcionadas aos problemas de saúde, fortalecendo as linhas de defesa e resistência. Ele demonstra potencial como referencial teórico nesse campo.

## INTRODUÇÃO

O Modelo de Sistemas de Neuman (MSN) é baseado na filosofia de que cada ser humano é um sistema aberto, afetado pelos ambientes externo e interno, de modo a garantir balanceamento e adaptação^(1)^. Ele se concentra na resposta das linhas de defesa normal e de resistência a estressores ambientais, bem como no uso de intervenções preventivas de enfermagem para promover, alcançar e manter o bem-estar ótimo do sistema do cliente^(2)^.

O núcleo do sistema do cliente é o foco central, cuja prioridade é manter essa estrutura básica livre de estresse. As linhas de resistência e de defesa protegem o sistema contra estressores com recursos das variáveis fisiológicas, psicológicas, sociológicas, desenvolvimentais e espirituais, que entram em ação quando um estressor rompe uma das linhas, comprometendo a estabilidade do sistema^(2)^.

Os estressores, contra os quais as linhas de defesa e de resistência protegem o sistema do cliente, podem ser intrapessoais (dentro do sistema do cliente), interpessoais (próximos aos limites do sistema do cliente) ou extrapessoais (distantes do sistema do cliente). Esses estressores podem ser múltiplos, e o seu impacto é afetado pelo tipo, tempo e reação do sistema do cliente, abrangendo as variáveis fisiológicas, psicológicas, sociológicas, desenvolvimentais ou espirituais^(3)^. As ações de prevenção, como intervenção primária, secundária e terciária, serão desenvolvidas de acordo com o tempo e o nível de severidade dos danos causados por esses estressores nas linhas de defesa e de resistência^(3)^. A Figura 1 traz uma representação gráfica do MSN.

**Figura 1:** Diagrama do modelo de sistemas de Neuman.

O uso do MSN é necessário para a testagem de vários fenômenos no campo da enfermagem nos ambientes cultural e social^(4)^. Nesse aspecto, associar o uso do MSN à área da saúde da mulher é algo promissor, pois essa área tem se expandido, saindo do foco no cuidado materno para incluir saúde sexual, contracepção, doenças crônicas específicas do contexto feminino, violência interpessoal, entre outros fenômenos^(5)^.

As mulheres são as que mais procuram os serviços de saúde e são as mais vulneráveis aos estigmas sociais impostos, às violências, aos padrões rígidos sociais e às fragilidades nos *status* social e econômico, estando, portanto, mais susceptíveis aos riscos de ter a saúde afetada^(6)^. É observado que, em diferentes momentos de sua vida, as mulheres estão expostas a estressores, que incluem fatores psicológicos, sociais e econômicos, que afetam significativamente os resultados da saúde das mulheres.

Nesse cenário, compreende-se a complexidade do atendimento em saúde da mulher. Logo, o profissional de enfermagem é muito importante ao disponibilizar atendimentos para a prevenção de doenças, a proteção e a promoção da saúde durante consultas de pré-natal, consultas de prevenção ginecológica, puerpério, planejamento familiar, entre outras demandas espontâneas^(7)^.

Assim, para um cuidado de enfermagem estruturado e eficaz voltado à saúde da mulher, o uso de teorias e modelos é crucial na translação do conhecimento de enfermagem gerado para a prática e pesquisa, visto que criam uma base sólida para a implementação e avaliação das intervenções, promovendo a integração entre o conhecimento teórico e as necessidades práticas^(8)^.

Considerando essas informações, o MSN é apropriado para o cuidado de forma integral de mulheres, pois propõe uma abordagem de sistema aberto, envolvendo cinco grandes variáveis. Ele pode ser aplicado a uma variedade de populações e condições, sendo singularmente adaptável a uma gama de questões de enfermagem. Além disso, pode ser utilizado para delinear papéis primários, secundários e terciários do enfermeiro junto ao sistema do cliente^(9,10)^.

Todavia, apesar do seu potencial explicativo e da aplicabilidade reconhecida, observa-se que a utilização do modelo na saúde da mulher carece de sistematização e aprofundamento crítico. Em muitos estudos, o referencial teórico é utilizado de maneira parcial, meramente descritiva ou ilustrativa, sem que haja a incorporação integral de seus elementos ou a explicitação de como esses elementos orientam a análise, a interpretação dos achados ou o planejamento de intervenções. Essa utilização fragmentada pode enfraquecer a consistência teórica das investigações e limitar o avanço cumulativo do conhecimento na área.

Foi realizada uma busca nas bases de dados *Cumulative Index to Nursing and Allied Health Literature* (CINAHL Plus), PubMed/MEDLINE e Scopus, bem como no portal Biblioteca Virtual em Saúde (BVS), além das fontes *JBI Evidence Synthesis, Cochrane Database of Systematic Reviews* e *Open Science Framework* (OSF) *Registry*, em 28 de abril de 2024. Entretanto, não foram encontradas revisões de escopo ou sistemáticas sobre o uso do MSN na área da saúde da mulher.

Diante desse cenário, torna-se imprescindível mapear criticamente as evidências científicas sobre o uso do MSN na saúde da mulher, analisando não apenas onde ele tem sido aplicado, mas também como seus elementos têm sido incorporados. Essa iniciativa contribui para o fortalecimento epistemológico da enfermagem para a qualificação da produção científica e para a promoção de um cuidado mais coerente, fundamentado e teoricamente consistente. Devido à abordagem holística do MSN e ao seu potencial para abordar problemas específicos da saúde da mulher e a escassez de estudos sobre sua aplicação nessa área, surgiu a seguinte questão: como o MSN tem sido utilizado na área da saúde da mulher?

Nesse contexto, tem-se como objetivo mapear as evidências científicas sobre o uso do MSN na área da saúde da mulher. Espera-se, com os resultados desta revisão, compreender a utilização do MSN nesse cenário.

## MÉTODO

### Desenho de Estudo

Trata-se de revisão de escopo conduzida de acordo com as diretrizes do Manual da JBI para síntese de evidências e relatada conforme as recomendações do *Preferred Reporting Items for Systematic review and Meta-Analyses extension for Scoping Reviews* ^(11,12)^. O protocolo desta revisão está disponível no OSF através do *link*: https://osf.io/zw52h/.

### Questão de Revisão

Para nortear o desenvolvimento da revisão, a questão de pesquisa foi estruturada a partir do acrônimo PCC, que significa População, Conceito e Contexto. Desse modo, a População foi considerada como em aberto; o Conceito inclui o MSN; e o Contexto inclui a saúde da mulher. Assim, surgiu a seguinte pergunta norteadora da revisão: como o MSN tem sido utilizado na área da saúde da mulher?

### Critérios de Inclusão

Foram incluídos os estudos que utilizaram o MSN, abordaram o contexto da saúde da mulher e foram publicados como pesquisas primárias, revisões de literatura, editoriais, artigos de opinião, cartas, comentários, livros, teses ou dissertações.

### Critérios de Exclusão

Foram excluídos os estudos cujos dados sobre a saúde das mulheres não foram tratados de forma separada das demais populações.

### Fontes de Informação

Foram consultadas as bases de dados Scopus (Elsevier), PubMed/MEDLINE, Embase, Ovid, *Web of Science*, CINAHL *with Full Text* e o portal BVS. Além disso, a literatura cinzenta foi consultada a partir da Biblioteca Digital Brasileira de Teses e Dissertações, do ProQuest para teses e dissertações internacionais e do *Google Scholar* (dez primeiras páginas).

Também foi realizada uma busca na lista de referências dos artigos incluídos na amostra final com o auxílio da ferramenta *online ResearchRabbit.*

### Estratégia de Busca

Antes das buscas nas bases de dados, foi realizada uma busca na PubMed/MEDLINE para definir os descritores utilizados na estratégia com a ajuda de um bibliotecário. Devido ao fato de o MSN não constar no *Medical Subject Headings* (MeSH), foram utilizados descritores não controlados para as bases de dados que utilizam esse vocabulário, baseados nos termos encontrados nos títulos, nos resumos e nas palavras-chave dos artigos identificados na busca preliminar. Os descritores referentes ao conceito “saúde da mulher” foram identificados no MeSH, juntamente com seus sinônimos. Para as demais bases de dados, foi construída uma estratégia de busca adaptada para os vocabulários específicos.

Descritores similares foram utilizados para a literatura cinzenta. As buscas foram realizadas sem restrições de idioma e anos de publicação. Os descritores de cada base de dados e fontes alternativas, bem como as estratégias de busca, podem ser consultados em seu conjunto no repositório SciELO Data, cujo link encontra-se ao final deste artigo. O Quadro 1 apresenta uma das estratégias utilizadas para busca na base de dados *Web of Science*.

**Quadro 1:** Estratégia de busca utilizada na base de dados *Web of Science* para revisão – Fortaleza, CE, 2024.

Base de dados	Estratégia de busca
*Web of Science*	*“Neuman Systems Model” OR “Betty Neuman Systems Model” OR “Neuman’s System Theory” OR “Neuman’s System” OR “Neuman’s Model” OR “Neuman’s framework” OR “Betty Neuman’s System” OR “Betty Neuman’s Systems Model” OR “Neuman systems theory” OR “Neumans System” OR “Neuman System’s” OR “Betty Neuman’s assumptions” OR “Betty Neuman Health Care Systems Model” OR “Betty Neuman” (All Fields) and “Women” OR “Women’s Health” OR “Reproductive Health” OR Contraception OR “Breast Neoplasms” OR “Health, Reproductive” OR “Inhibition of Fertilization” OR “Fertilization Inhibition” OR “Fertility Control” OR “Birth Control” OR “Contraceptive Methods” OR “Female Contraception” OR “Neoplasm, Breast” OR “Breast Tumors” OR “Mammary Cancer” OR “Malignant Neoplasm of Breast” OR “Cancer of Breast” OR “Human Mammary Carcinomas” OR “Human Mammary Neoplasm” OR “Human Mammary Neoplasms” OR “Breast Carcinoma” OR “Change of Life, Female” OR “Pregnancies” OR “Gestation” OR “Health, Sexual” (All Fields)*

### Seleção dos Estudos

Para o gerenciamento das referências obtidas nas bases de dados, foi utilizada a ferramenta *online Rayyan* nas fases de remoção de duplicatas, leitura de títulos e resumos e leitura na íntegra^(13)^.

A busca dos estudos nas bases de dados e na literatura cinzenta ocorreu no dia 29 de abril de 2024. O processo de seleção dos estudos compreendeu o período entre 30 de abril e 10 de junho de 2024. Primeiramente, foram removidas as duplicatas de artigos. Em seguida, foi feita a leitura de títulos e resumos. Os estudos remanescentes foram lidos na íntegra e avaliados criteriosamente, a fim de justificar sua inclusão ou exclusão da amostra final. As etapas de leitura de títulos e resumos e leitura na íntegra foram realizadas por dois revisores. Na fase de títulos e resumos, não houve conflitos, enquanto que, na leitura na íntegra, houve cinco conflitos, nos quais um terceiro revisor tomou a decisão final de incluir ou não os artigos. Um *checklist* eletrônico foi utilizado para nortear essas etapas de seleção (link para o repositório de dados ao final do manuscrito).

Após a inclusão dos estudos na amostra final, a ferramenta *online ResearchRabbit* foi utilizada para busca nas listas de referências, a fim de encontrar mais estudos que respondessem à pergunta da revisão.

### Extração dos Dados

Os dados foram extraídos por meio de um instrumento elaborado pelos autores, contendo as seguintes informações: autor(es); ano de publicação; país de origem (local em que a fonte foi conduzida); objetivos/propósito; população e tamanho da amostra dentro da fonte de evidência (se aplicável); características da população e critérios de inclusão e exclusão; métodos (tipo de estudo, cenário, duração, recrutamento/seleção, análise de dados); detalhes da intervenção e comparador (se aplicável); duração da intervenção (se aplicável); detalhes do instrumento utilizado (se aplicável); e resultados (se aplicável).

Além das características gerais, foram coletadas informações pertinentes ao objetivo do estudo, tais como: área da saúde da mulher abordada; variáveis abordadas (fisiológicas, psicológicas, socioculturais, desenvolvimentais e espirituais); ambiente (interno, externo e criado); fatores estressores (intrapessoais, interpessoais e extrapessoais); linhas de defesa afetadas (flexível, normal e de resistência); prevenção (primária, secundária e terciária); limitações do estudo; conclusões; e comentários dos revisores.

### Análise das Evidências

Os dados referentes às características gerais dos estudos foram analisados por meio de estatística descritivas (frequência absoluta e relativa). Os dados relativos ao objetivo do estudo foram analisados com base na estrutura do MSN e codificados de acordo com seus elementos, como variáveis identificadas, fatores estressores, linhas de defesa e tipos de prevenção aplicados como intervenção, bem como a forma como o modelo foi utilizado no estudo (avaliação, aporte teórico, intervenção *etc*.).

## RESULTADOS

Conforme apresentado na Figura 2, a busca nas bases de dados resultou em 343 estudos, dos quais 120 eram duplicatas e foram removidos. Os 223 restantes foram analisados por meio de títulos e resumos. Desses, 183 foram excluídos conforme os critérios pré-estabelecidos. Além disso, 17 estudos não tinham o texto completo disponível, seja por indisponibilidade de *link* para acesso ou compra, seja por site da revista desativado. Quando os dados estavam indisponíveis, foram feitas tentativas de contato com as revistas e os autores dos artigos por e-mail, mas não se obteve resposta até o momento. Desse modo, foram incluídos na revisão 23 estudos, sendo que um não estava relacionado à saúde da mulher e cinco não utilizaram, de fato, o MSN. Desse modo, 17 estudos foram incluídos na revisão. A busca na literatura cinzenta, no *Google Scholar* e na lista de referência dos artigos totalizou 557 estudos. Desses, 129 não eram relacionados à saúde da mulher e 426 não utilizaram o MSN. Portanto, dois estudos foram incluídos na revisão, somando-se 19 estudos que compõem a amostra final.

**Figura 2:** Fluxograma *PRISMA-ScR* de busca e seleção dos estudos para revisão^(12)^.

O Quadro 2 apresenta a caracterização dos estudos incluídos na revisão, de acordo com o tipo de estudo, a área da saúde da mulher abordada e os seus objetivos. Doze estudos foram desenvolvidos nos Estados Unidos, quatro, na Turquia, um, na Suécia, um, na Coreia do Sul, e um, em Portugal. Os desenhos metodológicos foram variados, predominando estudos com abordagem qualitativa (n = 5), estudos de intervenção (n = 3) e estudos transversais (n = 3). Uma versão expandida dessa tablea pode ser verificada no repositório de dados, cujo link está ao final do manuscrito, que contém detalhadamente a relação entre os aspectos estudados em cada artigo e os elementos do MSN.

**Quadro 2:** Caracterização dos estudos incluídos na revisão – Fortaleza, CE, 2024.

Autor, ano/país	Amostra	Tipo de estudo	Área da saúde da mulher	Objetivo
Aker e Özdemir, 2023/Turquia^(15)^	65 mulheres inférteis acima de 18 anos de idade	Ensaio clínico randomizado pré- e pós-teste	Inseminação artificial	Determinar o efeito da abordagem baseada no Modelo de Sistemas de Neuman no estresse e no enfrentamento em mulheres que recebem tratamento de inseminação intrauterina.
Bigbee, 1984/Estados Unidos^(16)^	Mulheres	Revisão narrativa	Papel materno	Revisar a literatura relevante sobre as mudanças de papéis das mulheres residentes nas áreas rurais dos Estados Unidos.
Bourdeanu e Dee, 2013/Estados Unidos^(17)^	Mulher	Estudo de caso fictício	Câncer de mama	Demonstrar o uso do Modelo de Sistemas de Neuman para avaliar e determinar os preditores de náuseas e vômitos induzidos por quimioterapia em pacientes com diagnóstico de câncer de mama.
Chaponniere, 2016/Estados Unidos^(18)^	84 gestantes de 16 a 41 anos de idade	Série de casos	Gestação e parto	Investigar a relação entre estresse, amortecedores de estresse, aculturação e resultados de nascimento de bebês nascidos de mães mexicanas e mexicano-americanas.
Doherty, 1997/Estados Unidos^(19)^	Seis mulheres	Estudo etnográfico e grupos focais	Violência doméstica/abuso conjugal	Explorar o abuso conjugal em famílias afro-americanas que vivem em Tucson por parte de mulheres vítimas de abuso para formar indutivamente um ponto de vista cultural.
Ïnan e Üstün, 2018/Turquia^(20)^	32 mulheres de 32 a 70 anos de idade	Quasi-experimental pré- e pós-teste	Câncer de mama	Avaliar os efeitos de um programa psicoeducacional domiciliar sobre sofrimento, ansiedade, depressão e qualidade de vida em sobreviventes de câncer de mama.
Ïnan e Üstün, 2016/Turquia^(21)^	14 mulheres de 33 a 68 anos	Qualitativo descritivo	Câncer de mama	Explicar as experiências dos sobreviventes turcas do câncer de mama à luz do Modelo de Sistemas de Neuman.
Gigliotti, 1999/Estados Unidos^(22)^	188 alunas de enfermagem	Transversal	Papel materno	Examinar as condições sob as quais o estresse do papel de mãe-estudante se desenvolve.
Gigliotti, 2007/Estados Unidos^(23)^	69 mães universitárias	Transversal	Papel materno	Melhorar a validade externa e interna do modelo de Gigliotti de estresse do papel materno-estudantil em mulheres de meia-idade.
Hemphill, 2005/Estados Unidos^(24)^	17 moradoras de rua de 22 a 56 anos de idade	Qualitativo descritivo	Violência	Envolver mulheres vítimas de violência em situação de rua em um diálogo que facilite a descoberta de sua percepção e de seus pontos fortes pessoais.
Kim et al., 2020/Coreia do Sul^(25)^	Mães solteiras	Revisão integrativa	Papel materno	Revisar de forma abrangente as pesquisas domésticas sobre mães solteiras com base nos principais conceitos que constituem o Modelo de Sistemas de Neuman.
Lancaster, 2005/Estados Unidos^(26)^	209 mulheres	Estudo descritivo correlacional	Câncer de mama	Examinar como as mulheres com histórico familiar de câncer de mama avaliam e lidam com o risco de câncer de mama.
Lindell e Olsson 1991/Suécia^(27)^	Parteiras	Estudo teórico	Contracepção	Explorar como a parteira sueca pode usar na prática o Modelo de Sistemas de Neuman no fornecimento de aconselhamento contraceptivo oral combinado.
Mataoui, 2018/Estados Unidos^(28)^	118 mulheres árabes acima de 40 anos de idade	Transversal	Câncer de mama	Avaliar o bem-estar do núcleo central por meio da identificação da taxa de triagem para mulheres árabes muçulmanas que vivem nos Estados Unidos com 40 anos ou mais e com 50 anos ou mais, e comparar os resultados com os dados dos Centros de Controle e Prevenção de Doenças, que são, respectivamente, 65,3% e 71,3%. Avaliar as inter-relações entre aculturação, modéstia e conhecimento sobre rastreamento de câncer de mama. Avaliar os efeitos do estressor no núcleo central.
Metin e Kulakaç, 2023/Turquia^(29)^	201 gestantes de alto risco	Estudo metodológico	Gestação	Desenvolver a Escala de Estresse Percebido em Gestações de Risco baseada no Modelo de Sistemas de Neuman.
Reeves, 2004/Estados Unidos^(30)^	Oito mulheres vítimas de violência	Qualitativo fenomenológico	Violência por parceiro íntimo	Compreender as experiências vividas na infância por mulheres vítimas de violência por parceiro íntimo.
Sabatini, 2003/Estados Unidos^(31)^	11 mulheres de 16 a 27 anos de idade	Qualitativo fenomenológico	Gestação	Explorar o significado da experiência vivida da gravidez na adolescência para mulheres que deram à luz na adolescência.
Sousa et al., 2022/Portugal^(32)^	Uma mulher de 53 anos de idade	Estudo de caso qualitativo	Câncer de mama	Compreender a vivência do câncer parental de uma mulher-mãe à luz do Modelo de Sistemas de Neuman, identificar as necessidades da mulher-mãe e prescrever intervenções de enfermagem recorrendo à taxonomia Classificação Internacional para a Prática de Enfermagem.
Tabone, 2013/Estados Unidos^(14)^	45 mulheres atletas universitárias de 18 a 22 anos de idade	Quasi-experimental pré- e pós-teste	Tríade da atleta feminina	Identificar os déficits de conhecimento e destacar a importância de educar atletas universitárias sobre a tríade da atleta feminina. Fornecer educação às atletas femininas sobre a prevalência da tríade das atletas femininas.

O câncer (CA) de mama e a maternidade (gestação, parto e papel materno) foram as áreas da saúde da mulher mais abordadas, com seis estudos cada, seguidos da violência (doméstica, por parceiro íntimo) (n = 3). Outras temáticas, como a inseminação artificial, a contracepção e a tríade da atleta feminina, também foram contempladas.

O MSN foi utilizado para melhorar ou avaliar os efeitos do estresse em procedimentos clínicos, na gestação e no papel materno. No contexto do CA de mama, as vivências das pacientes foram analisadas, incluindo aspectos como ansiedade, depressão e qualidade de vida. Além disso, os efeitos fisiológicos do tratamento quimioterápico (náuseas e vômitos) também foram abordados.

A compreensão do impacto da violência na vida diária e na infância, bem como seus aspectos culturais, a partir do MSN, também foi abordada nos estudos desta revisão. O modelo também foi utilizado em intervenções para aconselhamento contraceptivo realizado por parteiras e para educação em saúde relacionada à tríade da atleta feminina. A tríade da atleta feminina é uma síndrome composta por distúrbios alimentares, amenorreia e osteoporose. A síndrome pode se manifestar em atletas de alto rendimento com nutrição inadequada^(14)^.

Quanto aos elementos do MSN utilizados nos estudos, notou-se que a maioria (n = 14) usou alguma classificação das variáveis (fisiológica, psicológica, sociocultural, desenvolvimental e espiritual), e cinco (26,3%) não utilizaram (Quadro 3). Entre os que utilizaram, a maior prevalência foi das variáveis psicológicas, socioculturais e desenvolvimentais (85,8%), seguidas das variáveis fisiológicas (78,6%) e espirituais (71,4%).

**Quadro 3:** Elementos do Modelo de Sistemas de Neuman utilizados nos estudos – Fortaleza, CE, 2024.

Autor/ano	Variáveis	Estressores	Linhas de defesa/resistência	Prevenção
Aker e Özdemir, 2023^(15)^	Não classificou	Intrapessoais Interpessoais Extrapessoais	Não abordou	Secundária
Bigbee, 1984^(16)^	Não classificou	Intrapessoais Interpessoais Extrapessoais	LDF	Não abordou
Bourdeanu e Dee, 2013^(17)^	Fisiológica Psicológica Sociocultural Desenvolvimental Espiritual	Intrapessoais Interpessoais Extrapessoais	Não abordou	Primária Secundária Terciária
Chaponniere, 2016^(18)^	Fisiológica Psicológica Sociocultural Desenvolvimental Espiritual	Não classificou	LDF LDN LR	Não abordou
Doherty, 1997^(19)^	Não classificou	Intrapessoais Interpessoais Extrapessoais	LDF LR	Primária Secundária
Ïnan e Üstün, 2018^(20)^	Não classificou	Não classificou	LR	Terciária
Ïnan e Üstün, 2016^(21)^	Fisiológica Psicológica Sociocultural Desenvolvimental Espiritual	Intrapessoais Interpessoais Extrapessoais	Não abordou	Terciária
Gigliotti, 1999^(22)^	Fisiológica Psicológica Sociocultural Desenvolvimental	Intrapessoais Interpessoais	LDF LDN	Não abordou
Gigliotti, 2007^(23)^	Psicológica Sociocultural Desenvolvimental	Não classificou	LDF LN	Não abordou
Hemphill, 2005^(24)^	Psicológica Espiritual	Intrapessoais Interpessoais Extrapessoais	Não abordou	Não abordou
Kim et al., 2020^(25)^	Fisiológica Psicológica Sociocultural Desenvolvimental Espiritual	Intrapessoais Interpessoais Extrapessoais	LDF LDN LR	Não abordou
Lancaster, 2005^(26)^	Não classificou	Não classificou	LDF	Primária
Lindell e Olsson, 1991^(27)^	Fisiológica Psicológica Sociocultural Desenvolvimental Espiritual	Intrapessoais Interpessoais Extrapessoais	LDF	Primária
Mataoui, 2018^(28)^	Sociocultural Desenvolvimental	Não classificou	LDN	Não se aplica
Metin e Kulakaç, 2023^(29)^	Fisiológica Psicológica Sociocultural Desenvolvimental Espiritual	Não classificou	Não abordou	Não abordou
Reeves, 2004^(30)^	Fisiológica Psicológica Sociocultural Desenvolvimental Espiritual	Intrapessoais Interpessoais Extrapessoais	LDF LDN LR	Primária Secundária Terciária
Sabatini, 2003^(31)^	Fisiológica Psicológica Sociocultural Desenvolvimental Espiritual	Não classificou	Não abordou	Não abordou
Sousa et al., 2022^(32)^	Fisiológica Psicológica Sociocultural Desenvolvimental Espiritual	Intrapessoais Interpessoais Extrapessoais	LDF LR	Primária Secundária Terciária
Tabone, 2013^(14)^	Fisiológica	Não classificou	Não abordou	Primária

Legenda: LDF – linha de defesa flexível; LDN – linha de defesa normal; LR – linha de resistência.

Os estressores (intra, inter e extrapessoais) foram classificados em 11 estudos (58%). As linhas de defesa (flexível e normal) e de resistência foram abordadas em 12 estudos (63%); desses, três abordaram todas as linhas. A linha de defesa flexível, que é a primeira a entrar em contato com os estressores, foi a que mais frequentemente apareceu nos artigos que abordaram esse elemento (n = 7; 37%). A prevenção como intervenção (primária, secundária e terciária) foi realizada ou sugerida em dez artigos. Três deles contemplaram todos os níveis de prevenção, sendo a primária a mais frequente (n = 7; 37%). No entanto, deve-se levar em consideração que cada fenômeno possui níveis de prevenção mais adequados ao que está sendo trabalhado.

O ambiente, classificado como interno, externo e criado, foi identificado em dois estudos. No primeiro, um estudo qualitativo sobre mulheres vítimas de violência em situação de rua, o ambiente criado foi formado pela percepção delas sobre sua resposta pessoal aos estressores ambientais^(24)^. No segundo estudo, uma revisão integrativa sobre o papel da mãe solteira, o ambiente externo foi composto por apoio parental, apoio do pai da criança, apego dos adultos e apego dos pais^(25)^.

De maneira geral, o uso do MSN possibilitou o alcance de resultados positivos, como a redução dos níveis de estresse e dos mecanismos de enfrentamento, a melhor organização e o melhor planejamento do cuidado em enfermagem por meio de uma visão holística, a avaliação da relação entre as variáveis em estudo e a possibilidade de uso em intercâmbio com outras teorias^(15,17,21,22,30,32)^.

## DISCUSSÃO

Esta revisão proporcionou uma visão geral de como o MSN tem sido utilizado na saúde da mulher e a sua aplicabilidade prática e teórica. O câncer de mama, tema mais trabalhado, foi abordado sob a perspectiva da mulher, considerando o estresse e a ansiedade relacionados tanto ao diagnóstico quanto ao risco de desenvolver a doença. A identificação das variáveis envolvidas nessa problemática é importante para o planejamento do cuidado de enfermagem.

Não apenas os aspectos psicológicos, mas também os fisiológicos relacionados aos fatores do tratamento da doença foram associados aos estressores, como o tipo de tratamento, outras condições médicas, outros problemas (por exemplo, fadiga), hábitos de vida, uso de antidepressivos e falta de preparação para lidar com sintomas persistentes^(21)^. Isso sugere que o modelo pode ser aplicado tanto em situações subjetivas, em estudos qualitativos, quanto em situações objetivas, em estudos quantitativos.

O processo de ser mãe, incluindo a gestação, o parto e o papel materno, foi outro aspecto bastante trabalhado com o MSN, especificamente no que se refere ao estresse e à ansiedade inerentes a esse papel social e ao momento de vida. Nota-se que o modelo é adequado para avaliar situações complexas que envolvem múltiplas variáveis, como no caso do papel materno-estudantil, em que os papéis se sobrepõem, e os efeitos de estressores interpessoais são de extrema importância^(22)^. O mesmo foi observado em um estudo sobre o significado da gravidez na adolescência, cujos autores afirmam que o MSN fornece estrutura e organização a um tópico que, de outra forma, seria extremamente complexo^(31)^.

A violência, outro tema complexo, foi explorada sob a perspectiva cultural e de condição social. Entender, a partir do MSN, como esse fenômeno impactou a vida das mulheres que participaram dos estudos trouxe à tona o papel do enfermeiro ao planejar intervenções para restaurar a estabilidade do sistema dessas clientes.

Os elementos do MSN foram abordados de diversas maneiras. Ao avaliar os efeitos de um programa psicoeducacional domiciliar no sofrimento, na ansiedade, na depressão e na qualidade de vida em sobreviventes de CA de mama, verificou-se que, ao fortalecer as linhas de resistência por meio da prevenção terciária, houve uma redução do estresse psicológico e uma melhora na qualidade de vida dessas pacientes^(20)^.

Um estudo que avaliou a violência contra mulheres evidenciou que a prevenção terciária seria a intervenção mais apropriada, pois os fatores de estresse anteriores causaram perturbações no sistema. Isso pode ser alcançado por meio de apoio social e de informações educacionais que abordem o impacto físico e psicológico a longo prazo da violência praticada pelo parceiro íntimo^(30)^.

Já a prevenção primária pode ser utilizada em temas que são relacionados à educação em saúde, como o aconselhamento contraceptivo, cujo objetivo é prevenir os riscos de um fator estressor ameaçar o núcleo, como uma gravidez indesejada, e fortalecer a linha flexível de defesa^(27)^. Para isso, é necessário que as percepções do cliente sejam avaliadas antes do desenvolvimento de uma intervenção específica. A intervenção deve diminuir o estresse do sistema e aumentar os fatores positivos individuais e coletivos da sua saúde, fortalecendo o sistema do cliente^(24)^.

Percebe-se que os elementos do MSN são aplicáveis à saúde da mulher em situações variadas. No entanto, poucos estudos detiveram-se a explorar o ambiente em suas análises. É importante que esse elemento seja levado em consideração, visto que a forma como a mulher expressa o ambiente criado depende dos recursos percebidos que ela traz para uma experiência particular, e a percepção criada por uma mulher sobre uma experiência pode influenciar seu estado de saúde^(24)^.

Considerando-se a ampla gama de situações e os variados desenhos metodológicos em que o MSN foi utilizado no contexto da saúde da mulher, verifica-se que a implementação do modelo pelos enfermeiros pode aumentar a eficácia dos cuidados, identificando os problemas e favorecendo as mudanças positivas^(21)^. Além disso, o modelo permite que a participante se torne um membro ativo no processo do cuidar, em que a autopercepção e a autoavaliação são vistas como aspectos essenciais para alcançar e manter o bem-estar^(31)^.

Os estudos apontaram, também, os benefícios da visão holística da mulher proporcionada pelo modelo. Neuman afirma que a prática de enfermagem é holística e que incluir a percepção de saúde do cliente é parte integrante da prestação de cuidados. Por meio desse modelo, todo o processo de saúde é abordado, incluindo a promoção da saúde, os cuidados agudos, crônicos e de fim de vida^(24,30)^. A determinação dos níveis existentes de recursos energéticos positivos disponíveis na defesa contra estressores auxilia a mulher na busca pelo bem-estar. Cabe aos enfermeiros avaliar esses recursos energéticos e utilizá-los para desenvolver intervenções que minimizem o impacto dos estressores no sistema, promovendo a restauração da saúde no nível mais elevado possível^(24)^.

O modelo tem potencial para ser utilizado como referencial teórico nas áreas da saúde da mulher abordadas nesta revisão e, possivelmente, em outras que não apareceram como resultados obtidos neste estudo. A saúde da mulher é multidimensional, e um modelo que considere não só os aspectos individuais da cliente, mas também suas relações interpessoais e fatores extrapessoais, oferece uma visão holística. Essa abordagem amplia as possibilidades de intervenção para os enfermeiros que desejam trabalhar com esse público, permitindo um cuidado mais abrangente e eficaz.

### Limitações do Estudo

Um considerável número de estudos não pôde ser recuperado por se tratarem de teses ou dissertações indisponíveis, conforme a política das instituições responsáveis por seus repositórios.

## CONCLUSÃO

O MSN tem sido utilizado na Saúde da Mulher como estrutura para avaliar e intervir na saúde de mulheres com CA de mama; avaliar o estresse relacionado ao papel materno; diminuir estresse e ansiedade relacionados aos procedimentos; entender o impacto de diferentes formas de violência; desenvolver prevenções primárias como educação em saúde para aconselhamento contraceptivo; e esclarecer sobre condições inerentes à saúde feminina. A abordagem utilizada com o MSN nos estudos é majoritariamente qualitativa, tratando de temas complexos e subjetivos que envolvem múltiplos elementos e necessitam de avaliação aprofundada por parte do enfermeiro.

Embora seja um modelo holístico, poucos estudos utilizaram todos os elementos em sua estrutura. Entre as variáveis, as mais utilizadas foram as psicológicas, socioculturais e desenvolvimentais. Os estressores, as linhas de defesa e as prevenções como intervenção foram abordados em proporções similares em pouco mais da metade dos estudos. O ambiente, por outro lado, foi o elemento menos abordado.

A escolha por não utilizar todos os elementos do modelo pode ter relação com a situação abordada em cada estudo, pois os autores podem não ter achado necessário o seu uso de forma completa. O baixo foco no uso do ambiente nos estudos pode estar relacionado a uma visão mais focada no problema do que no contexto, o que pode ter subestimado a dimensão dos aspectos analisados em estudos que não utilizaram este elemento. Isso ressalta a necessidade de mais estudos na área da saúde da mulher, utilizando sua potencialidade para melhorar sua saúde, incluindo o ambiente externo e interno, e garantindo que o núcleo do sistema do cliente esteja livre ou seja menos exposto ao estresse.

O MSN, como demonstrado no estudo, tem uma perspectiva holística e integral que visa não somente as necessidades individuais, mas todo o contexto em que a mulher está envolvida. A partir de seus elementos, o modelo pode ser aplicado integralmente ou em situações específicas, proporcionando uma visão ampla e facilitando a identificação de pontos de intervenção para atingir a estabilização do sistema do cliente. Essa aplicação pode se dar através de análise da situação com o próprio modelo e de teorias de médio alcance ou teorias de situação específica derivadas do MSN, visto que é uma teoria de abstração mais elevada.

Este estudo reforça o potencial do MSN como estrutura organizadora do cuidado, favorecendo intervenções direcionadas à redução de estressores e ao fortalecimento dos mecanismos de proteção da mulher, alinhando-se a uma perspectiva sistêmica e integral da saúde da mulher. Além disso, ao identificar as áreas já exploradas e os contextos ainda pouco investigados, o estudo contribui para a definição de agendas futuras de pesquisa, estimulando investigações que utilizem o modelo de forma mais robusta e que ampliem sua aplicação em políticas públicas, na Atenção Primária e em estratégias de promoção da saúde.

## Data Availability

Todo o conjunto de dados que dá suporte aos resultados deste estudo encontra-se no repositório SciELO Data através do link: https://doi.org/10.48331/SCIELODATA.DP0S60.

# Translation: Uso del Modelo de Sistemas de Neuman en la salud de la mujer: revisión de alcance

**DOI:** 10.1590/1980-220X-REEUSP-2025-0460es

**Authors:** Nathaly Bianka Moraes Fróes, Dayane dos Reis Araújo Rocha Holanda, Samila Gomes Ribeiro, Paula Renata Amorim Lessa Soares, Francisca Elisângela Teixeira Lima, Viviane Martins da Silva, Marcos Venícios de Oliveira Lopes

**Affiliations:** 1 Universidade Federal do Ceará, Fortaleza, CE, Brasil.

**Keywords:** Modelos Teóricos, Salud de la Mujer, Teoría de Enfermería, Revisión de Alcance

## Abstract

**Objetivo:**

Mapear la evidencia científica sobre el uso del Modelo de Sistemas de Neuman (MSN) en la salud de la mujer.

**Método:**

Revisión del alcance. La búsqueda se realizó de abril a junio de 2024 en las siguientes bases de datos: Scopus, PubMed, Embase, Ovid, Web of Science, CINAHL, Biblioteca Virtual en Salud, Biblioteca Digital Brasileña de Tesis y Disertaciones, ProQuest y Google Scholar. Se incluyeron investigaciones primarias, revisiones, editoriales, artículos de opinión, cartas, comentarios y literatura gris que utilizaron el modelo en el ámbito de la salud femenina.

**Resultados:**

El modelo se utilizó para evaluar el estrés relacionado con procedimientos y la maternidad, comprender las experiencias de pacientes con cáncer de mama, analizar la violencia, ofrecer asesoramiento sobre anticoncepción y educar sobre salud en la tríada de la atleta femenina. Los elementos del modelo se identificaron en cada escenario estudiado, pero pocos los utilizaron por completo.

**Conclusión:**

El MSN ha proporcionado un marco de acción en salud femenina, utilizando las cinco variables para diseñar intervenciones dirigidas a problemas de salud, fortaleciendo las líneas de defensa y resistencia. Demuestra potencial como marco teórico en este campo.

## INTRODUCCIÓN

El Modelo de Sistemas de Neuman (MSN) se basa en la filosofía de que cada ser humano es un sistema abierto, afectado por los entornos externo e interno, con el fin de garantizar el equilibrio y la adaptación^(1)^. Se centra en la respuesta de las líneas de defensa normales y de resistencia ante los factores estresantes ambientales, así como en el uso de intervenciones preventivas de enfermería para promover, alcanzar y mantener el bienestar óptimo del sistema del cliente^(2)^.

El núcleo del sistema del cliente es el punto central, cuya prioridad es mantener esta estructura básica libre de estrés. Las líneas de resistencia y de defensa protegen al sistema contra los factores estresantes mediante recursos de las variables fisiológicas, psicológicas, sociológicas, de desarrollo y espirituales, que entran en acción cuando un factor estresante rompe una de las líneas, comprometiendo la estabilidad del sistema^(2)^.

Los factores estresantes, contra los cuales las líneas de defensa y resistencia protegen al sistema del cliente, pueden ser intrapersonales (dentro del sistema del cliente), interpersonales (cercanos a los límites del sistema del cliente) o extrapersonales (distantes del sistema del cliente).

Estos factores estresantes pueden ser múltiples, y su impacto depende del tipo, el momento y la reacción del sistema del cliente, abarcando variables fisiológicas, psicológicas, sociológicas, de desarrollo o espirituales^(3)^. Las medidas de prevención, como la intervención primaria, secundaria y terciaria, se desarrollarán de acuerdo con el tiempo y el nivel de gravedad de los daños causados por estos factores estresantes en las líneas de defensa y resistencia^(3)^. La Figura 1 presenta una representación gráfica del MSN.

**Figura 1:** Diagrama del modelo de sistemas de Neuman.

El uso del MSN es necesario para analizar diversos fenómenos en el campo de la enfermería en los entornos cultural y social^(4)^. En este sentido, asociar el uso del MSN al ámbito de la salud de la mujer resulta prometedor, ya que esta área se ha expandido, pasando de centrarse en la atención materna a incluir la salud sexual, la anticoncepción, las enfermedades crónicas específicas del contexto femenino y la violencia interpersonal, entre otros fenómenos^(5)^.

Las mujeres son las que más recurren a los servicios de salud y son las más vulnerables a los estigmas sociales impuestos, a la violencia, a los patrones sociales rígidos y a las fragilidades en su *estatus* social y económico, por lo que son más susceptibles a los riesgos de que su salud se vea afectada^(6)^. Se observa que, en diferentes momentos de su vida, las mujeres están expuestas a factores estresantes —que incluyen aspectos psicológicos, sociales y económicos— que afectan significativamente sus resultados de salud.

En este contexto, se comprende la complejidad de la atención en salud de la mujer. Por lo tanto, el profesional de enfermería es muy importante al brindar servicios para la prevención de enfermedades, la protección y la promoción de la salud durante las consultas prenatales, las consultas de prevención ginecológica, el posparto y la planificación familiar, entre otras demandas espontáneas^(7)^.

Así, para una atención de enfermería estructurada y eficaz orientada a la salud de la mujer, el uso de teorías y modelos es crucial para trasladar el conocimiento de enfermería generado a la práctica y la investigación, ya que crean una base sólida para la implementación y evaluación de las intervenciones, promoviendo la integración entre el conocimiento teórico y las necesidades prácticas^(8)^.

Teniendo en cuenta esta información, el MSN es adecuado para la atención integral de las mujeres, ya que propone un enfoque de sistema abierto que involucra cinco variables principales. Se puede aplicar a una variedad de poblaciones y condiciones, y es singularmente adaptable a una amplia gama de cuestiones de enfermería. Además, puede utilizarse para definir los roles primarios, secundarios y terciarios del enfermero en relación con el sistema del cliente^(9,10)^.

Sin embargo, a pesar de su potencial explicativo y de su reconocida aplicabilidad, se observa que el uso del modelo en la salud de la mujer carece de sistematización y de un análisis crítico más profundo. En muchos estudios, el marco teórico se utiliza de manera parcial, meramente descriptiva o ilustrativa, sin que se incorporen íntegramente sus elementos ni se explique cómo estos orientan el análisis, la interpretación de los hallazgos o la planificación de las intervenciones. Este uso fragmentado puede debilitar la consistencia teórica de las investigaciones y limitar el avance acumulativo del conocimiento en el área.

Se realizó una búsqueda en las bases de datos *Cumulative Index to Nursing and Allied Health Literature* (CINAHL Plus), PubMed/MEDLINE y Scopus, así como en el portal Biblioteca Virtual en Salud (BVS), además de las fuentes *JBI Evidence Synthesis, Cochrane Database of Systematic Reviews* y *Open Science Framework* (OSF) *Registry*, el 28 de abril de 2024.

Sin embargo, no se encontraron revisiones de alcance ni sistemáticas sobre el uso del MSN en el ámbito de la salud de la mujer.

Ante este panorama, resulta imprescindible realizar un mapeo crítico de la evidencia científica sobre el uso del MSN en la salud de la mujer, analizando no solo dónde se ha aplicado, sino también cómo se han incorporado sus elementos. Esta iniciativa contribuye al fortalecimiento epistemológico de la enfermería para la cualificación de la producción científica y para la promoción de una atención más coherente, fundamentada y teóricamente consistente. Debido al enfoque holístico de la MSN y a su potencial para abordar problemas específicos de la salud de la mujer, así como a la escasez de estudios sobre su aplicación en esta área, surgió la siguiente pregunta: ¿cómo se ha utilizado la MSN en el ámbito de la salud de la mujer?

En este contexto, el objetivo es mapear la evidencia científica sobre el uso del MSN en el ámbito de la salud de la mujer. Se espera que, con los resultados de esta revisión, se comprenda la utilización del MSN en este escenario.

## MÉTODO

### Diseño del Estudio

Se trata de una revisión de alcance realizada de acuerdo con las directrices del Manual de la JBI para la síntesis de evidencia y reportada conforme a las recomendaciones de los *Preferred Reporting Items for Systematic reviews and Meta-Analyses: extension for Scoping Reviews* ^(11,12)^. El protocolo de esta revisión está disponible en la OSF a través del *enlace*: https://osf.io/zw52h/.

### Pregunta de Investigación

Para orientar el desarrollo de la revisión, la pregunta de investigación se estructuró a partir del acrónimo PCC, que significa Población, Concepto y Contexto. De este modo, la Población se consideró abierta; el Concepto incluye el MSN; y el Contexto incluye la salud de la mujer. Así, surgió la siguiente pregunta orientadora de la revisión: ¿cómo se ha utilizado el MSN en el ámbito de la salud de la mujer?

### Criterios de Inclusión

Se incluyeron los estudios que utilizaron el MSN, abordaron el contexto de la salud de la mujer y se publicaron como investigaciones primarias, revisiones de literatura, editoriales, artículos de opinión, cartas, comentarios, libros, tesis o disertaciones.

### Criterios de Exclusión

Se excluyeron los estudios cuyos datos sobre la salud de las mujeres no se trataron de manera separada del resto de las poblaciones.

### Fuentes de Información

Se consultaron las bases de datos Scopus (Elsevier), PubMed/MEDLINE, Embase, Ovid, *Web of Science*, CINAHL *with Full Text* y el portal BVS. Además, se consultó la literatura gris a través de la Biblioteca Digital Brasileña de Tesis y Disertaciones, de ProQuest para tesis y disertaciones internacionales y de *Google Scholar* (las primeras diez páginas).

También se realizó una búsqueda en la lista de referencias de los artículos incluidos en la muestra final con la ayuda de la herramienta *en línea ResearchRabbit.*

### Estrategia de Búsqueda

Antes de realizar las búsquedas en las bases de datos, se llevó a cabo una búsqueda en PubMed/MEDLINE para definir los descriptores utilizados en la estrategia con la ayuda de un bibliotecario. Debido a que «MSN» no figura en *Medical Subject Headings* (MeSH), se utilizaron descriptores no controlados para las bases de datos que emplean este vocabulario, basados en los términos encontrados en los títulos, los resúmenes y las palabras clave de los artículos identificados en la búsqueda preliminar.

Los descriptores relacionados con el concepto «salud de la mujer» se identificaron en el MeSH, junto con sus sinónimos. Para el resto de las bases de datos, se elaboró una estrategia de búsqueda adaptada a los vocabularios específicos.

Se utilizaron descriptores similares para la literatura gris. Las búsquedas se realizaron sin restricciones de idioma ni de años de publicación. Los descriptores de cada base de datos y fuentes alternativas, así como las estrategias de búsqueda, pueden consultarse en su conjunto en el repositorio SciELO Data, cuyo enlace se encuentra al final de este artículo. El Cuadro 1 presenta una de las estrategias utilizadas para la búsqueda en la base de datos *Web of Science*.

**Cuadro 1:** Estrategia de búsqueda utilizada en la base de datos *Web of Science* para la revisión – Fortaleza, CE, 2024.

Base de datos	Estrategia de búsqueda
*Web of Science*	*“Neuman Systems Model” OR “Betty Neuman Systems Model” OR “Neuman’s System Theory” OR “Neuman’s System” OR “Neuman’s Model” OR “Neuman’s framework” OR “Betty Neuman’s System” OR “Betty Neuman’s Systems Model” OR “Neuman systems theory” OR “Neumans System” OR “Neuman System’s” OR “Betty Neuman’s assumptions” OR “Betty Neuman Health Care Systems Model” OR “Betty Neuman” (All Fields) and “Women” OR “Women’s Health” OR “Reproductive Health” OR Contraception OR “Breast Neoplasms” OR “Health, Reproductive” OR “Inhibition of Fertilization” OR “Fertilization Inhibition” OR “Fertility Control” OR “Birth Control” OR “Contraceptive Methods” OR “Female Contraception” OR “Neoplasm, Breast” OR “Breast Tumors” OR “Mammary Cancer” OR “Malignant Neoplasm of Breast” OR “Cancer of Breast” OR “Human Mammary Carcinomas” OR “Human Mammary Neoplasm” OR “Human Mammary Neoplasms” OR “Breast Carcinoma” OR “Change of Life, Female” OR “Pregnancies” OR “Gestation” OR “Health, Sexual” (All Fields)*

### Selección de los Estudios

Para la gestión de las referencias obtenidas en las bases de datos, se utilizó la herramienta *en línea Rayyan* en las etapas de eliminación de duplicados, lectura de títulos y resúmenes, y lectura completa^(13)^.

La búsqueda de los estudios en las bases de datos y en la literatura gris se realizó el 29 de abril de 2024. El proceso de selección de los estudios abarcó el período comprendido entre el 30 de abril y el 10 de junio de 2024. En primer lugar, se eliminaron los artículos duplicados. A continuación, se procedió a la lectura de los títulos y resúmenes. Los estudios restantes se leyeron en su totalidad y se evaluaron minuciosamente, con el fin de justificar su inclusión o exclusión de la muestra final. Las etapas de lectura de títulos y resúmenes, así como la lectura completa, fueron realizadas por dos revisores. En la fase de títulos y resúmenes no hubo desacuerdos, mientras que, en la lectura completa, se presentaron cinco desacuerdos, en los que un tercer revisor tomó la decisión final de incluir o no los artículos. Se utilizó una *lista de verificación* electrónica para guiar estas etapas de selección (link para el repositorio de dados al final del manuscrito).

Tras la inclusión de los estudios en la muestra final, se utilizó la herramienta *en línea ResearchRabbit* para realizar búsquedas en las listas de referencias, con el fin de encontrar más estudios que respondieran a la pregunta de la revisión.

### Extracción de Datos

Los datos se extrajeron mediante un instrumento elaborado por los autores, que contenía la siguiente información: autor(es); año de publicación; país de origen (lugar donde se llevó a cabo el estudio); objetivos/propósito; población y tamaño de la muestra dentro de la fuente de evidencia (si corresponde); características de la población y criterios de inclusión y exclusión; métodos (tipo de estudio, escenario, duración, reclutamiento/selección, análisis de datos); detalles de la intervención y el comparador (si corresponde); duración de la intervención (si corresponde); detalles del instrumento utilizado (si corresponde); y resultados (si corresponde).

Además de las características generales, se recopiló información pertinente al objetivo del estudio, tal como: área de la salud de la mujer abordada; variables abordadas (fisiológicas, psicológicas, socioculturales, de desarrollo y espirituales); entorno (interno, externo y creado); factores estresantes (intrapersonales, interpersonales y extrapersonales); líneas de defensa afectadas (flexible, normal y de resistencia); prevención (primaria, secundaria y terciaria); limitaciones del estudio; conclusiones; y comentarios de los revisores.

### Análisis de la Evidencia

Los datos relativos a las características generales de los estudios se analizaron mediante estadísticas descriptivas (frecuencia absoluta y relativa). Los datos relativos al objetivo del estudio se analizaron basándose en la estructura del MSN y se codificaron de acuerdo con sus elementos, tales como variables identificadas, factores estresantes, líneas de defensa y tipos de prevención aplicados como intervención, así como la forma en que se utilizó el modelo en el estudio (evaluación, aporte teórico, intervención *etc*.).

## RESULTADOS

Como se muestra en la Figura 2, la búsqueda en las bases de datos arrojó 343 estudios, de los cuales 120 eran duplicados y fueron eliminados. Los 223 restantes se analizaron a partir de los títulos y resúmenes. De estos, 183 se excluyeron según los criterios preestablecidos. Además, 17 estudios no tenían el texto completo disponible, ya sea por la falta de un *enlace* para acceder o comprarlo, o porque el sitio web de la revista estaba inactivo. Cuando los datos no estaban disponibles, se intentó contactar a las revistas y a los autores de los artículos por correo electrónico, pero hasta el momento no se ha obtenido respuesta. De este modo, se incluyeron en la revisión 23 estudios, de los cuales uno no estaba relacionado con la salud de la mujer y cinco, de hecho, no utilizaron el MSN. Así, se incluyeron 17 estudios en la revisión. La búsqueda en la literatura gris, en *Google Scholar* y en la lista de referencias de los artículos arrojó un total de 557 estudios. De estos, 129 no estaban relacionados con la salud de la mujer y 426 no utilizaron el MSN. Por lo tanto, se incluyeron dos estudios en la revisión, lo que suma un total de 19 estudios que conforman la muestra final.

**Figura 2:** Diagrama de flujo *PRISMA-ScR* de búsqueda y selección de los estudios para la revisión^(12)^.

El Cuadro 2 presenta la caracterización de los estudios incluidos en la revisión, según el tipo de estudio, el área de salud de la mujer abordada y sus objetivos. Doce estudios se realizaron en Estados Unidos, cuatro en Turquía, uno en Suecia, uno en Corea del Sur y uno en Portugal. Los diseños metodológicos fueron variados, predominando los estudios con enfoque cualitativo (n = 5), los estudios de intervención (n = 3) y los estudios transversales (n = 3). En el repositorio de dados, cuyo link está al final del manucrito, se puede consultar una versión ampliada de esta tabla, que detalla la relación entre los aspectos estudiados en cada artículo y los elementos del MSN.

**Cuadro 2:** Características de los estudios incluidos en la revisión – Fortaleza, CE, 2024.

Autor, año/país	Muestra	Tipo de estudio	Área de la salud de la mujer	Objetivo
Aker y Özdemir, 2023/Turquía^(15)^	65 mujeres infértiles mayores de 18 años	Ensayo clínico aleatorizado con pruebas previas y posteriores	Inseminación artificial	Determinar el efecto del enfoque basado en el Modelo de Sistemas de Neuman sobre el estrés y la capacidad de afrontamiento en mujeres que reciben tratamiento de inseminación intrauterina.
Bigbee, 1984/Estados Unidos^(16)^	Mujeres	Revisión narrativa	Papel materno	Revisar la bibliografía relevante sobre los cambios en los roles de las mujeres residentes en zonas rurales de Estados Unidos.
Bourdeanu y Dee, 2013/Estados Unidos^(17)^	Mujer	Estudio de caso ficticio	Cáncer de mama	Demostrar el uso del Modelo de Sistemas de Neuman para evaluar y determinar los factores predictivos de las náuseas y los vómitos inducidos por la quimioterapia en pacientes con diagnóstico de cáncer de mama.
Chaponniere, 2016/Estados Unidos^(18)^	84 mujeres embarazadas de entre 16 y 41 años	Serie de casos	Embarazo y parto	Investigar la relación entre el estrés, los factores amortiguadores del estrés, la aculturación y los resultados del parto en bebés nacidos de madres mexicanas y mexicoamericanas.
Doherty, 1997/Estados Unidos^(19)^	Seis mujeres	Estudio etnográfico y grupos focales	Violencia doméstica/maltrato conyugal	Explorar el maltrato conyugal en familias afroamericanas que viven en Tucson desde la perspectiva de las mujeres víctimas de maltrato para construir inductivamente una visión cultural.
Ïnan y Üstün, 2018/Turquía^(20)^	32 mujeres de entre 32 y 70 años	Diseño cuasi-experimental con pruebas previas y posteriores	Cáncer de mama	Evaluar los efectos de un programa psicoeducativo a domicilio sobre el sufrimiento, la ansiedad, la depresión y la calidad de vida en supervivientes de cáncer de mama.
Ïnan y Üstün, 2016/Turquía^(21)^	14 mujeres de entre 33 y 68 años	Estudio cualitativo descriptivo	Cáncer de mama	Explicar las experiencias de las supervivientes turcas de cáncer de mama a la luz del Modelo de Sistemas de Neuman.
Gigliotti, 1999/Estados Unidos^(22)^	188 estudiantes de enfermería	Transversal	Papel materno	Analizar las condiciones en las que se desarrolla el estrés derivado del papel de madre-estudiante.
Gigliotti, 2007/Estados Unidos^(23)^	69 madres universitarias	Transversal	Papel materno	Mejorar la validez externa e interna del modelo de Gigliotti sobre el estrés relacionado con el papel materno-estudiantil en mujeres de mediana edad.
Hemphill, 2005/Estados Unidos^(24)^	17 mujeres sin hogar de entre 22 y 56 años	Cualitativo descriptivo	Violencia	Involucrar a mujeres víctimas de violencia que viven en la calle en un diálogo que facilite el descubrimiento de su percepción y de sus puntos fuertes personales.
Kim et al., 2020/Corea del Sur^(25)^	Madres solteras	Revisión integrativa	Papel materno	Revisar de forma exhaustiva las investigaciones nacionales sobre madres solteras basándose en los conceptos principales que conforman el Modelo de Sistemas de Neuman.
Lancaster, 2005/Estados Unidos^(26)^	209 mujeres	Estudio descriptivo correlacional	Cáncer de mama	Analizar cómo las mujeres con antecedentes familiares de cáncer de mama evalúan y afrontan el riesgo de padecer esta enfermedad.
Lindell y Olsson, 1991/Suecia^(27)^	Comadronas	Estudio teórico	Anticoncepción	Explorar cómo las comadronas suecas pueden aplicar en la práctica el Modelo de Sistemas de Neuman a la hora de ofrecer a l s asesoramiento sobre anticonceptivos orales combinados.
Mataoui, 2018/Estados Unidos^(28)^	118 mujeres árabes mayores de 40 años	Estudio transversal	Cáncer de mama	Evaluar el bienestar del núcleo central mediante la identificación de la tasa de cribado en mujeres árabes musulmanas que viven en Estados Unidos con 40 años o más y con 50 años o más, y comparar los resultados con los datos de los Centros para el Control y la Prevención de Enfermedades, que son, respectivamente, del 65,3 % y del 71,3 %. Evaluar las interrelaciones entre la aculturación, la modestia y el conocimiento sobre el cribado del cáncer de mama. Evaluar los efectos del factor estresante en el núcleo central.
Metin y Kulakaç, 2023/Turquía^(29)^	201 embarazadas de alto riesgo	Estudio metodológico	Embarazo	Desarrollar la Escala de Estrés Percibido en Embarazos de Riesgo basada en el Modelo de Sistemas de Neuman.
Reeves, 2004/Estados Unidos^(30)^	Ocho mujeres víctimas de violencia	Cualitativo fenomenológico	Violencia por parte de la pareja	Comprender las experiencias vividas en la infancia por mujeres víctimas de violencia por parte de la pareja.
Sabatini, 2003/Estados Unidos^(31)^	11 mujeres de entre 16 y 27 años	Cualitativo fenomenológico	Embarazo	Explorar el significado de la experiencia vivida del embarazo en la adolescencia para las mujeres que dieron a luz durante la adolescencia.
Sousa et al., 2022/Portugal^(32)^	Una mujer de 53 años	Estudio de caso cualitativo	Cáncer de mama	Comprender la experiencia del cáncer parental de una mujer-madre a la luz del Modelo de Sistemas de Neuman, identificar las necesidades de la mujer-madre y prescribir intervenciones de enfermería recurriendo a la taxonomía de la Clasificación Internacional para la Práctica de Enfermería.
Tabone, 2013/Estados Unidos^(14)^	45 mujeres deportistas universitarias de entre 18 y 22 años	Estudio cuasi-experimental con pruebas previas y posteriores	Tríada de la deportista femenina	Identificar las lagunas de conocimiento y destacar la importancia de educar a las deportistas universitarias sobre la tríada de la deportista femenina. Impartir formación a las deportistas femeninas sobre la prevalencia de la tríada de la deportista femenina.

El cáncer de mama y la maternidad (gestación, parto y rol materno) fueron las áreas de salud de la mujer más abordadas, con seis estudios cada una, seguidas por la violencia (doméstica, por pareja íntima) (n = 3). También se abordaron otros temas, como la inseminación artificial, la anticoncepción y la tríada de la deportista.

El MSN se utilizó para mejorar o evaluar los efectos del estrés en procedimientos clínicos, durante la gestación y en el rol materno. En el contexto del CA de mama, se analizaron las experiencias de las pacientes, incluyendo aspectos como la ansiedad, la depresión y la calidad de vida. Además, también se abordaron los efectos fisiológicos del tratamiento quimioterapéutico (náuseas y vómitos).

La comprensión del impacto de la violencia en la vida cotidiana y en la infancia, así como sus aspectos culturales, a partir del MSN, también se abordó en los estudios de esta revisión. El modelo también se utilizó en intervenciones de asesoramiento anticonceptivo realizadas por parteras y para la educación en salud relacionada con la tríada de la deportista. La tríada de la deportista femenina es un síndrome compuesto por trastornos alimentarios, amenorrea y osteoporosis. El síndrome puede manifestarse en atletas de alto rendimiento con una nutrición inadecuada^(14)^.

En cuanto a los elementos del MSN utilizados en los estudios, se observó que la mayoría (n = 14) utilizó alguna clasificación de las variables (fisiológica, psicológica, sociocultural, de desarrollo y espiritual), y cinco (26,3 %) no la utilizaron (Cuadro 3). Entre los que las utilizaron, la mayor prevalencia correspondió a las variables psicológicas, socioculturales y de desarrollo (85,8 %), seguidas de las variables fisiológicas (78,6 %) y espirituales (71,4 %).

**Cuadro 3:** Elementos del Modelo de Sistemas de Neuman utilizados en los estudios – Fortaleza, CE, 2024.

Autor/año	Variables	Factores estresantes	Líneas de defensa/resistencia	Prevención
Aker y Özdemir,2023^(15)^	No clasificado	Intrapersonales Interpersonales Extrapersonales	No lo abordó	Secundaria
Bigbee, 1984^(16)^	No clasificó	Intrapersonales Interpersonales Extrapersonales	LDF	No se ha abordado
Bourdeanu y Dee, 2013^(17)^	Fisiológica Psicológica Sociocultural Del desarrollo Espiritual	Intrapersonales Interpersonales Extrapersonales	No se ha abordado	Primaria Secundaria Tercia
Chaponniere, 2016^(18)^	Fisiológica Psicológica Sociocultural Del desarrollo Espiritual	Sin clasificar	LDF LDN LR	No se ha abordado
Doherty, 1997^(19)^	No clasificó	Intrapersonales Interpersonales Extrapersonales	LDF LR	Primaria Secundaria
Ïnan y Üstün, 2018^(20)^	Sin clasificar	Sin clasificar	LR	Educación superior
Ïnan y Üstün, 2016^(21)^	Fisiológica Psicológica Sociocultural Del desarrollo Espiritual	Intrapersonales Interpersonales Extrapersonales	No se ha abordado	Tercia
Gigliotti, 1999^(22)^	Fisiológica Psicológica Sociocultural De desarrollo	Intrapersonales Interpersonales	LDF LDN	No se ha abordado
Gigliotti, 2007^(23)^	Psicológica Sociocultural Del desarrollo	No clasificó	LDF LN	No se ha abordado
Hemphill, 2005^(24)^	Psicológica Espiritual	Intrapersonales Interpersonales Extrapersonales	No se abordó	No se ha abordado
Kim et al., 2020^(25)^	Fisiológica Psicológica Sociocultural Del desarrollo Espiritual	Intrapersonales Interpersonales Extrapersonales	LDF LDN LR	No se ha abordado
Lancaster, 2005^(26)^	Sin clasificar	Sin clasificar	LDF	Primaria
Lindell y Olsson, 1991^(27)^	Fisiológica Psicológica Sociocultural Del desarrollo Espiritual	Intrapersonales Interpersonales Extrapersonales	LDF	Primaria
Mataoui, 2018^(28)^	Sociocultural De desarrollo	Sin clasificar	LDN	No aplicable
Metin y Kulakaç, 2023^(29)^	Fisiológica Psicológica Sociocultural Del desarrollo Espiritual	No clasificó	No se ha abordado	No se ha abordado
Reeves, 2004^(30)^	Fisiológica Psicológica Sociocultural Del desarrollo Espiritual	Intrapersonales Interpersonales Extrapersonales	LDF LDN LR	Primaria Secundaria Tercia
Sabatini, 2003^(31)^	Fisiológica Psicológica Sociocultural Del desarrollo Espiritual	No clasificó	No se ha abordado	No se ha abordado
Sousa et al., 2022^(32)^	Fisiológica Psicológica Sociocultural Del desarrollo Espiritual	Intrapersonales Interpersonales Extrapersonales	LDF LR	Primaria Secundaria Tercia
Tabone, 2013^(14)^	Fisiológica	Sin clasificar	No se ha abordado	Primaria

Leyenda: LDF – línea de defensa flexible; LDN – línea de defensa normal; LR – línea de resistencia.

Los factores estresantes (intra, inter y extrapersonales) se clasificaron en 11 estudios (58 %). Las líneas de defensa (flexible y normal) y de resistencia se abordaron en 12 estudios (63 %); de ellos, tres abordaron todas las líneas. La línea de defensa flexible, que es la primera en entrar en contacto con los factores estresantes, fue la que apareció con mayor frecuencia en los artículos que abordaron este elemento (n = 7; 37 %). La prevención como intervención (primaria, secundaria y terciaria) se llevó a cabo o se sugirió en diez artículos. Tres de ellos contemplaron todos los niveles de prevención, siendo la primaria la más frecuente (n = 7; 37 %). No obstante, hay que tener en cuenta que cada fenómeno presenta niveles de prevención más adecuados en función de lo que se esté trabajando.

El entorno, clasificado en interno, externo y creado, se identificó en dos estudios. En el primero, un estudio cualitativo sobre mujeres víctimas de violencia que vivían en la calle, el entorno creado se formó a partir de la percepción que ellas tenían de su respuesta personal a los factores estresantes ambientales^(24)^. En el segundo estudio, una revisión integrativa sobre el papel de la madre soltera, el entorno externo se compuso del apoyo parental, el apoyo del padre del niño, el apego de los adultos y el apego de los padres^(25)^.

En general, el uso de la MSN permitió alcanzar resultados positivos, como la reducción de los niveles de estrés y de los mecanismos de afrontamiento, una mejor organización y planificación de la atención de enfermería a través de una visión holística, la evaluación de la relación entre las variables en estudio y la posibilidad de su uso en diálogo con otras teorías^(15,17,21,22,30,32)^.

## DISCUSIÓN

Esta revisión ha proporcionado una visión general de cómo se ha utilizado el MSN en la salud de la mujer y de su aplicabilidad práctica y teórica. El cáncer de mama, tema más abordado, se trató desde la perspectiva de la mujer, considerando el estrés y la ansiedad relacionados tanto con el diagnóstico como con el riesgo de desarrollar la enfermedad. La identificación de las variables involucradas en esta problemática es importante para la planificación de la atención de enfermería.

No solo los aspectos psicológicos, sino también los fisiológicos relacionados con los factores del tratamiento de la enfermedad se asociaron a los factores estresantes, como el tipo de tratamiento, otras afecciones médicas, otros problemas (por ejemplo, la fatiga), los hábitos de vida, el uso de antidepresivos y la falta de preparación para lidiar con síntomas persistentes^(21)^. Esto sugiere que el modelo puede aplicarse tanto en situaciones subjetivas, en estudios cualitativos, como en situaciones objetivas, en estudios cuantitativos.

El proceso de ser madre, incluyendo la gestación, el parto y el rol materno, fue otro aspecto ampliamente abordado con el MSN, específicamente en lo que se refiere al estrés y la ansiedad inherentes a ese rol social y a esa etapa de la vida. Cabe señalar que el modelo es adecuado para evaluar situaciones complejas que involucran múltiples variables, como en el caso del papel materno-estudantil, en el que los roles se superponen y los efectos de los factores estresantes interpersonales son de extrema importancia^(22)^. Lo mismo se observó en un estudio sobre el significado del embarazo en la adolescencia, cuyos autores afirman que el MSN brinda estructura y organización a un tema que, de otra manera, sería extremadamente complejo^(31)^.

La violencia, otro tema complejo, se exploró desde la perspectiva cultural y de la condición social. Comprender, a partir del MSN, cómo este fenómeno impactó la vida de las mujeres que participaron en los estudios puso de relieve el papel del personal de enfermería al planificar intervenciones para restablecer la estabilidad del sistema de estas usuarias.

Los elementos del MSN se abordaron de diversas maneras. Al evaluar los efectos de un programa psicoeducativo a domicilio sobre el sufrimiento, la ansiedad, la depresión y la calidad de vida en sobrevivientes de cáncer de mama, se observó que, al fortalecer las líneas de resistencia mediante la prevención terciaria, hubo una reducción del estrés psicológico y una mejora en la calidad de vida de estas pacientes^(20)^.

Un estudio que evaluó la violencia contra las mujeres puso de manifiesto que la prevención terciaria sería la intervención más adecuada, ya que los factores de estrés previos causaron alteraciones en el sistema. Esto puede lograrse mediante el apoyo social y la información educativa que aborde el impacto físico y psicológico a largo plazo de la violencia ejercida por la pareja íntima^(30)^.

Por su parte, la prevención primaria puede utilizarse en temas relacionados con la educación en salud, como el asesoramiento anticonceptivo, cuyo objetivo es prevenir los riesgos de que un factor estresante amenace la unidad familiar —como un embarazo no deseado— y fortalecer la línea flexible de defensa^(27)^. Para ello, es necesario evaluar las percepciones de la persona antes de desarrollar una intervención específica. La intervención debe reducir el estrés del sistema y aumentar los factores positivos, tanto individuales como colectivos, de su salud, fortaleciendo así el sistema de la clienta^(24)^.

Se observa que los elementos del MSN son aplicables a la salud de la mujer en diversas situaciones. Sin embargo, pocos estudios se han detenido a explorar el entorno en sus análisis. Es importante que se tome en cuenta este elemento, ya que la forma en que la mujer expresa el entorno creado depende de los recursos percibidos que aporta a una experiencia particular, y la percepción que una mujer tiene de una experiencia puede influir en su estado de salud^(24)^.

Teniendo en cuenta la amplia gama de situaciones y los diversos diseños metodológicos en los que se ha utilizado el MSN en el contexto de la salud de la mujer, se observa que la implementación del modelo por parte del personal de enfermería puede aumentar la eficacia de la atención, identificando los problemas y favoreciendo los cambios positivos^(21)^. Además, el modelo permite que la participante se convierta en un miembro activo del proceso de atención, en el que la autopercepción y la autoevaluación se consideran aspectos esenciales para alcanzar y mantener el bienestar^(31)^.

Los estudios también señalaron los beneficios de la visión holística de la mujer que ofrece el modelo. Neuman afirma que la práctica de enfermería es holística y que incluir la percepción de salud de la usuaria es parte integral de la prestación de cuidados. A través de este modelo, se aborda todo el proceso de salud, incluyendo la promoción de la salud, los cuidados agudos, crónicos y al final de la vida^(24,30)^. La determinación de los niveles existentes de recursos energéticos positivos disponibles para la defensa contra los factores estresantes ayuda a la mujer en su búsqueda del bienestar. Corresponde a las enfermeras evaluar estos recursos energéticos y utilizarlos para desarrollar intervenciones que minimicen el impacto de los factores estresantes en el sistema, promoviendo la restauración de la salud al nivel más alto posible^(24)^.

El modelo tiene el potencial de utilizarse como marco teórico en las áreas de la salud de la mujer abordadas en esta revisión y, posiblemente, en otras que no aparecieron como resultados obtenidos en este estudio. La salud de la mujer es multidimensional, y un modelo que considere no solo los aspectos individuales de la usuaria, sino también sus relaciones interpersonales y los factores extrapersonales, ofrece una visión holística. Este enfoque amplía las posibilidades de intervención para las enfermeras que desean trabajar con este público, permitiendo una atención más integral y eficaz.

### Limitaciones del Estudio

No se pudo recuperar un número considerable de estudios debido a que se trataba de tesis o disertaciones no disponibles, de acuerdo con la política de las instituciones responsables de sus repositorios.

## CONCLUSIÓN

La maestría en enfermería (MSN) se ha utilizado en el ámbito de la salud de la mujer como marco para evaluar e intervenir en la salud de las mujeres con cáncer de mama; evaluar el estrés relacionado con el rol materno; reducir el estrés y la ansiedad relacionados con los procedimientos; comprender el impacto de diferentes formas de violencia; desarrollar medidas de prevención primaria, como la educación en salud para el asesoramiento anticonceptivo; y brindar información sobre afecciones inherentes a la salud femenina. El enfoque utilizado con el MSN en los estudios es mayoritariamente cualitativo, y aborda temas complejos y subjetivos que involucran múltiples elementos y requieren una evaluación a fondo por parte del personal de enfermería.

Aunque se trata de un modelo holístico, pocos estudios han utilizado todos los elementos de su estructura. Entre las variables, las más utilizadas fueron las psicológicas, socioculturales y de desarrollo. Los factores estresantes, las líneas de defensa y las medidas preventivas como intervención se abordaron en proporciones similares en poco más de la mitad de los estudios. El entorno, por otro lado, fue el elemento menos abordado.

La decisión de no utilizar todos los elementos del modelo puede estar relacionada con la situación abordada en cada estudio, ya que es posible que los autores no hayan considerado necesario su uso completo. La escasa atención prestada al entorno en los estudios puede estar relacionada con una visión más centrada en el problema que en el contexto, lo que puede haber subestimado la dimensión de los aspectos analizados en los estudios que no utilizaron este elemento. Esto resalta la necesidad de más estudios en el área de la salud de la mujer, aprovechando su potencial para mejorar su salud, incluyendo el entorno externo e interno, y asegurando que el núcleo del sistema de la clienta esté libre o sea menos expuesto al estrés.

El MSN, como se demostró en el estudio, tiene una perspectiva holística e integral que apunta no solo a las necesidades individuales, sino a todo el contexto en el que se encuentra la mujer. A partir de sus elementos, el modelo puede aplicarse de manera integral o en situaciones específicas, proporcionando una visión amplia y facilitando la identificación de puntos de intervención para lograr la estabilización del sistema de la usuaria. Esta aplicación puede realizarse mediante el análisis de la situación con el propio modelo y con teorías de alcance medio o teorías de situaciones específicas derivadas del MSN, dado que se trata de una teoría de mayor nivel de abstracción.

Este estudio refuerza el potencial del MSN como estructura organizadora de la atención, favoreciendo intervenciones dirigidas a la reducción de los factores estresantes y al fortalecimiento de los mecanismos de protección de la mujer, alineándose con una perspectiva sistémica e integral de la salud de la mujer. Además, al identificar las áreas ya exploradas y los contextos aún poco investigados, el estudio contribuye a la definición de futuras agendas de investigación, estimulando estudios que utilicen el modelo de manera más sólida y que amplíen su aplicación en políticas públicas, en la atención primaria y en estrategias de promoción de la salud.

## Data Availability

Todo el conjunto de datos que respalda los resultados de este estudio se encuentra en el repositorio SciELO Data a través del enlace https://doi.org/10.48331/SCIELODATA.DP0S60.
